# Genomic occupancy of Runx2 with global expression profiling identifies a novel dimension to control of osteoblastogenesis

**DOI:** 10.1186/gb-2014-15-3-r52

**Published:** 2014-03-21

**Authors:** Hai Wu, Troy W Whitfield, Jonathan A R Gordon, Jason R Dobson, Phillip W L Tai, Andre J van Wijnen, Janet L Stein, Gary S Stein, Jane B Lian

**Affiliations:** 1 Department of Biochemistry, University of Vermont College of Medicine and Vermont Cancer Center, 89 Beaumont Avenue, Burlington, VT 05405, USA; 2 Department of Cell & Developmental Biology, University of Massachusetts Medical School, 364 Plantation Street, Worcester, MA 01655, USA; 3 Current address: Center for Computational Molecular Biology, Department of Molecular Biology, Cell Biology & Biochemistry, Brown University, 115 Waterman Street, Providence, RI 02912, USA; 4 Current address: Department of Computer Science, Brown University, 115 Waterman Street, Providence, RI 02912, USA; 5 Current address: Departments of Orthopedic Surgery and Biochemistry & Molecular Biology, Mayo Clinic, Medical Sciences Building 3-69, 200 First Street SW, Rochester, MN 55905, USA

## Abstract

**Background:**

Osteogenesis is a highly regulated developmental process and continues during the turnover and repair of mature bone. Runx2, the master regulator of osteoblastogenesis, directs a transcriptional program essential for bone formation through genetic and epigenetic mechanisms. While individual Runx2 gene targets have been identified, further insights into the broad spectrum of Runx2 functions required for osteogenesis are needed.

**Results:**

By performing genome-wide characterization of Runx2 binding at the three major stages of osteoblast differentiation - proliferation, matrix deposition and mineralization - we identify Runx2-dependent regulatory networks driving bone formation. Using chromatin immunoprecipitation followed by high-throughput sequencing over the course of these stages, we identify approximately 80,000 significantly enriched regions of Runx2 binding throughout the mouse genome. These binding events exhibit distinct patterns during osteogenesis, and are associated with proximal promoters and also non-promoter regions: upstream, introns, exons, transcription termination site regions, and intergenic regions. These peaks were partitioned into clusters that are associated with genes in complex biological processes that support bone formation. Using Affymetrix expression profiling of differentiating osteoblasts depleted of Runx2, we identify novel Runx2 targets including Ezh2, a critical epigenetic regulator; Crabp2, a retinoic acid signaling component; Adamts4 and Tnfrsf19, two remodelers of the extracellular matrix. We demonstrate by luciferase assays that these novel biological targets are regulated by Runx2 occupancy at non-promoter regions.

**Conclusions:**

Our data establish that Runx2 interactions with chromatin across the genome reveal novel genes, pathways and transcriptional mechanisms that contribute to the regulation of osteoblastogenesis.

## Background

The development of bone tissue, new bone formation in the adult, mineral homeostasis and maintenance of bone mass are mediated by cells of the osteoblast lineage. These bone forming cells progress through a highly regulated differentiation program, with each subpopulation of cells acquiring stage-specific phenotypes that are characterized by distinct profiles of expressed genes [1]. Osteoblast commitment and differentiation are dependent on the appropriate expression of Runx2 (Runt-related transcription factor 2), the master regulator of bone formation [1-3]. Ablation of Runx2 in mice results in the absence of a mineralized skeleton, and disruption of Runx2 function causes bone defects in the human disorder cleidocranial dysplasia [4,5]. Runx2 controls a complex gene-regulatory network during osteoblastogenesis [5,6]. It upregulates a variety of osteoblast lineage-specific genes, including *Osx* (osterix), *Ocn* (osteocalcin), and *Bsp* (bone sialoprotein), and represses the expression of non-osteoblast genes such as *PPAR-γ* (peroxisome proliferator-activated receptor gamma) and *MyoD* (myogenic differentiation), which are required for adipogenic and myogenic commitment, respectively [7-9]. Studies have demonstrated that Runx2 controls gene expression by interacting with multiple classes of co-regulatory factors [1,3,10]. In addition to the traditional transcriptional mechanisms, several epigenetic mechanisms have been identified for Runx2-mediated gene expression. For example, Runx2 is retained on mitotic chromosomes as an epigenetic bookmarking factor to maintain cellular identity after cell division [11]. A significant body of evidence substantiates the contribution of Runx2 towards the epigenetic regulation of gene expression during osteoblast differentiation, through interactions with histone deacetylases [12], histone acetyltransferases [13], and SWI/SNF complex components [14].

Runx2 has been shown to activate or repress gene expression through binding to *cis*-regulatory DNA elements, the Runx motif (TGTGGT) located in or near gene promoter regions [15]. Genome-wide binding profiles of the transcriptional factor CTCF and lineage-specific transcription factors, such as PPAR-γ, MyoD, and GATA3 (GATA binding protein 3), via ChIP-Seq (chromatin immunoprecipitation followed by high-throughput sequencing) have helped to delineate *cis*-regulatory networks that are critical for cell lineage control in adipocytes, myocytes and T cells, respectively [16-19]. These studies have highlighted the regulatory importance of long-range interactions and binding of phenotypic transcription factors to non-promoter genomic elements. These findings have greatly expanded existing paradigms of transcriptional regulation. In contrast, the entire scope of Runx2 binding elements remains largely unknown, hindering a comprehensive understanding of the *cis*-regulatory network through which Runx2 regulates the transcription program for bone formation.

Hence, we have characterized the genome-wide occupancy of Runx2 by ChIP-Seq in MC3T3-E1 preosteoblasts, a well-studied *in vitro* model for osteoblastogenesis [20]. Runx2 occupancy was determined at three hallmark stages of osteoblast differentiation: proliferation, matrix deposition, and mineralization. Analyses of these data demonstrated that Runx2 occupancy on genes is differentiation stage-dependent in both binding intensities and binding regions, indicating a shift in the regulatory mechanisms required for the entire program of osteoblastogenesis. By coupling genome-wide Runx2 binding with gene expression profiling, we have identified new Runx2 targets that were validated for functional activities of Runx2 binding in both promoter and non-promoter regions. Our study of Runx2 genome-wide occupancy establishes a foundation for future investigation of the Runx2-controlled regulatory network during bone formation and homeostasis.

## Results

### Runx2 dynamically occupies a wide range of genomic loci during osteoblastogenesis

**Figure 1:**
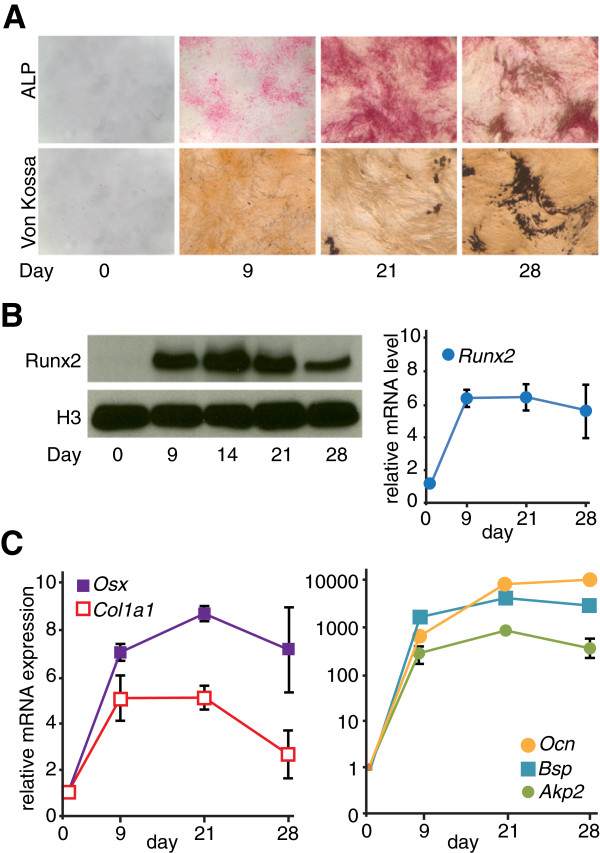
**The differentiation stages of murine MC3T3-E1 preosteoblasts used for profiling studies. (A)** Staining for alkaline phosphatase (ALP) activity (upper panel) and mineralization (Von Kossa, lower panel) in MC3T3-E1 cells during three stages of differentiation: proliferation (day 0), matrix deposition (days 9 to 21), and mineralization (day 28). **(B)** Protein (left panel) and mRNA levels (right panel) of *Runx2* during osteoblastic differentiation of MC3T3-E1 cells. Histone H3 (H3) was used as loading control for western blotting. **(C)** Expression profile of osteoblast-related markers *Osx/Sp7*, *Col1a1* (left panel), *Akp2/Alpl* (alkaline phosphatase liver/bone/kidney), *Bsp/Ibsp*, and *Ocn/Bglap2* (right panel) in MC3T3-E1 cells during differentiation. Relative mRNA levels (versus day 0) were determined by quantitative RT-PCR (reverse transcription PCR), normalized by *Hprt1* mRNA levels and plotted as mean values ± SEM (standard error of mean) from three independent biological replicates. The expression levels of the genes in **(B,C)** at days 9, 21, and 28 were significantly upregulated when compared to those at day 0 (*P* < 0.05, *t*-test).

Runx2 is a known master activator of bone formation, but thus far only a small number of osteoblast-specific target genes have been characterized [2,5,6]. To identify genome-wide occupancy of Runx2 in osteoblast lineage cells, we performed ChIP-Seq using a Runx2-specific antibody at stages during the *in vitro* differentiation of MC3T3-E1 preosteoblasts (Figure 1). This *in vitro* cell model recapitulates *in vivo* osteoblast differentiation and therefore was used for ChIP-Seq studies [21]. Alkaline phosphatase activity, an early osteoblast marker, increased as cells proceeded from proliferation (day 0) to matrix deposition (days 9 and 21) and decreased upon mineralization (day 28), visualized by Von Kossa staining (Figure 1A). Runx2 mRNA and protein levels significantly increased during the initial stage of differentiation. While the mRNA levels reached steady state, Runx2 protein levels declined during late mineralization (Figure 1B). Osteoblast phenotypic markers, including the transcription factor Osx/Sp7 (osterix/Sp7 transcription factor), a marker of committed osteoprogenitors, the extracellular matrix protein Col1a1 (collagen type I alpha 1), and the specialized mineral binding proteins Bsp/Ibsp (bone sialoprotein/integrin-binding sialoprotein) and Ocn/Bglap2 (osteocalcin/bone gamma-carboxyglutamate (gla) protein 2), displayed expression patterns consistent with the progression of osteoblastogenesis [2] (Figure 1C).

**Figure 2:**
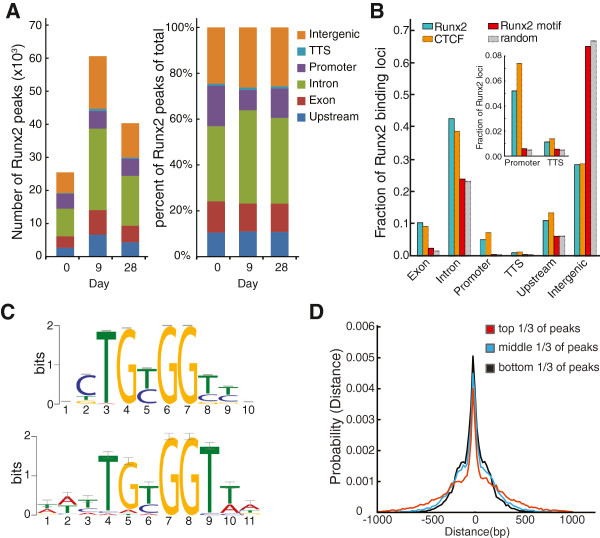
**Genome-wide profile of Runx2 occupancy. (A)** Distribution of Runx2 binding peaks across the mouse genome were classified into six categories of genomic locations: exon, intron, promoter (-1 kb to +150 bp of TSS), upstream (-1 kb to -20 kb from TSS), TTS region (-150 bp to -1 kb of transcription termination site), and intergenic region. Peak distribution was plotted at each time point by peak number (left panel) and by the percentage of total peaks (right panel). **(B)** Differential Runx2 enrichment in the six categories of genomic locations compared to the predicted Runx2 motif (see below), to random binding, or to binding of the transcription factor CTCF. Inset provides a magnified view of promoter and TTS regions. **(C)** The 500 most significant Runx2 peaks, based on MACS [22] significance (*P* < 1 × 10^-10^) were used for *de novo* motif discovery. A Runx2 motif (position weight matrix, top) with strong statistical confidence (*P* = 4.7 × 10^-200^) was determined using MEME (MEME suite version 4.7.0) [24]. The known Runx motif (MA0002.2, bottom) in JASPAR [26] was used for comparison using TOMTOM [24]. As shown in **(B)**, the distribution of *de novo* Runx2 motifs among categories of genomic locations was determined using FIMO [24] at a significance threshold of *P* < 10^-4^. **(D)** Probability plot of the distribution of Runx2 peaks indicating the distances of Runx2 motifs to the peak centers in the top, middle, and bottom third of Runx2 peaks (ranked by MACS scores) versus the probabilities of finding *de novo* Runx2 motifs at given positions relative to peak center.

We next isolated and sequenced DNA from chromatin bound by Runx2. Sequence reads from Runx2 ChIPs and input controls were mapped to the mouse genome. Statistically significant enrichments of Runx2 were identified by MACS (Model-Based Analysis of ChIP-Seq) [22] (Additional file 1). RefSeq annotations [23] were used to assign Runx2 enrichments to categories of genomic locations (with non-overlapping definitions for transcriptional start site (TSS), promoter, exonic, intronic, transcription termination site (TTS), upstream and intergenic regions (see Figure 2 for details; Additional file 2). As Runx2 protein levels changed, the total number of Runx2 peaks changed accordingly (Figure 2A, left panel). The overall distribution of Runx2 binding among the categories of genomic locations was relatively unchanged during differentiation (Figure 2A, right panel). Among genomic locations, Runx2 occupancy at promoters showed the greatest variation from 17.6% at day 0 to 8.8% at day 9 (Figure 2A, right panel). The majority of Runx2 binding occurred at intergenic and intronic regions (Figure 2A,B). However, when compared with a randomly sampled background distribution of genomic intervals (Additional file 3), Runx2 binding displayed preferential enrichment in a genic context, particularly at promoters and exons. One exception was under-represented Runx2 binding at intergenic regions when compared to nonspecific or random binding (Figure 2B). This relative enrichment of Runx2 occupancy in genic contexts was also observed in comparison with Runx2 motifs (Figure 2B). A *de novo* Runx2 motif (Figure 2C, top) was discovered using MEME [24] on the highest-ranked (by *P*-value) 500 ChIP-Seq peaks and was then scanned using FIMO [24] (version 4.7.0) over the mouse genome. Notably, analysis by MEME did not discover a significantly enriched secondary motif associated with Runx2 peaks. Despite the nearly random distribution of Runx2 motifs throughout the genome, Runx2 occupancy in differentiating osteoblasts is characterized by associations with promoters, exons, introns and other genic elements. These associations are perhaps due to epigenetic factors, including chromatin conformation and accessibility, along with co-factors, and suggest that the presence of a Runx motif does not necessarily indicate the physical association of Runx2. Interestingly, the distribution of Runx2 occupancy among classes of genomic elements is similar to that of CTCF (Figure 2B), a ubiquitous transcription factor that exhibits a broad spectrum of DNA binding in many cell lines [25].

The *de novo* Runx2 motif (Figure 2C, top) was scanned over all ChIP-Seq peaks called by MACS [22] from ChIP-Seq reads collected from day 9 MC3T3 cells, and a histogram of the distance between the peak summit and the highest-scoring motif instance was collected. The distribution in Figure 2D is characterized by a sharp peak for Runx2 motifs at the summits of ChIP-Seq peaks. Using TOMTOM [24] it was determined that the *de novo* Runx2 motif was similar (E-value = 2.6 × 10^-4^, q-value = 2.6 × 10^-4^) to the JASPAR MA0002.2 RUNX motif [26] (Figure 2C, bottom) and both motifs share the core TGTGGT sequence with the known Runx2 binding consensus.

### Genome-wide Runx2 occupancy reveals distinct positional and temporal binding patterns

**Figure 3:**
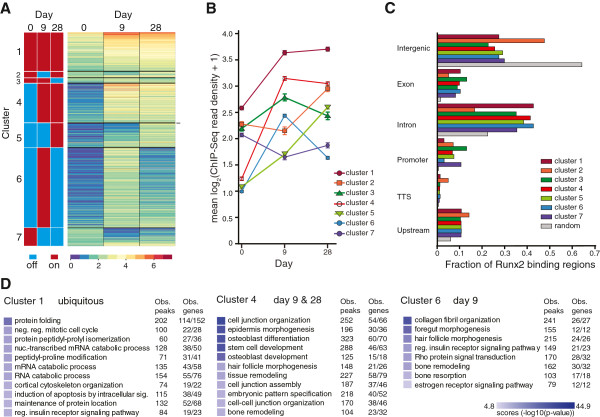
**Characterization of the temporal patterns of Runx2 binding associated with osteoblastogenesis. (A)** Enriched regions of Runx2 binding (MACS peaks, *P* ≤ 1 × 10^-10^) were grouped into seven clusters based on the presence (red) or absence (blue) of peaks at temporal stages of osteoblastic differentiation (left panel). In the right panel, each line illustrates intensity (base-2 logarithm of the sum of read counts per 10 million reads (mean read density)) of one peak as a range from strong occupancy (brown) to weak occupancy (violet). **(B)** Runx2 binding intensities of Runx2 peaks from each cluster at days 0, 9, and 28 of differentiation plotted as mean ± SEM. **(C)** Distribution patterns of the Runx2 peaks from each of the seven clusters (from **(A)**) segregated into six categories of genomic locations and compared to an equivalent number of random 100 bp regions (gray bar). **(D)** Representative Gene Ontology term annotation of cluster 4 (days 9 & 28) by GREAT. Each term is annotated with the observed (Obs.) number of Runx2 peaks and corresponding number of genes out of the total number of genes.

We grouped Runx2 peaks based on the presence or absence of Runx2 binding at specific time points during differentiation (Figure 3A, off/on). The resulting seven distinct clusters reflected the dynamics of Runx2 binding in relation to the progression of osteoblastogenesis (Figure 3A). In Figure 3B, the mean ChIP-Seq read densities (that is, peak intensities) of the clustered Runx2 peaks were plotted to compare their relative enrichments.

The seven clusters differed in the numbers and intensities of Runx2 peaks (Figure 3A). The largest group was cluster 6, which exhibited the presence of peaks primarily at day 9. This finding is consistent with day 9 representing committed osteoblasts with the highest amount of cellular Runx2 protein (Figure 1B) and therefore the greatest number of Runx2 peaks (Figure 3B). The strongest Runx2 peak intensities were found in cluster 1 reflecting regions that were bound by Runx2 constitutively throughout osteoblast differentiation (Figure 3B). The weakest Runx2 peak intensities among the three time points occurred at the peaks in cluster 7, with marginal enrichment of Runx2 at day 0. It is noteworthy that the peaks in cluster 4 have the second highest peak intensities at both days 9 and 28 (matrix deposition and mineralization stages; Figure 3B). Cluster 5, like clusters 1 and 4, exhibited the highest peak intensities on day 28, indicating their importance in maintaining osteoblast phenotype.

Runx2 binding in each cluster was further examined for distribution preferences of peaks in different genomic regions, in contrast to the genome-wide distribution of random 100 bp DNA fragments (detailed in Additional file 3; Figure 3C). The random DNA fragments (grey bars in Figure 3C) are distributed mainly in intergenic, intronic, and upstream regions. When compared to the random control, we observed that the distribution of Runx2-enriched peaks was biased towards gene regions (exons, introns, promoters, TTS regions, upstream regions). In contrast, Runx2 binding in the intergenic regions is lower than the random control (Figure 3C). In promoters and exons, all clusters showed the highest enrichment over random binding, suggesting strong regulation by Runx2 at these genomic regions.

To further explore the relationship between Runx2 peaks and peak-associated genes in osteogenic differentiation, we performed functional annotations for the peaks in the seven clusters using GREAT (Genomic Regions Enrichment of Annotations Tool; Figure 3D). Gene Ontology (GO) terms associated with the largest clusters are shown in Figure 3D. Runx2 binding in cluster 1 yielded GO terms of general biological processes such as protein folding and RNA metabolism (detailed in Additional file 4). Cluster 4 peaks at days 9 and 28 were frequently related to the GO terms of osteoblast differentiation, bone developmental processes, and osteogenic signaling pathways. These terms often included differentiation-related and well-known Runx2 target genes, such as *Runx2*, *Bsp/Ibsp*, and *Osx/Sp7* (Additional file 4). Similarly, cluster 6 (day 9) peaks often associated with bone formation and extracellular matrix organization. The annotation of other smaller clusters is shown in Additional file 5. For examples, cluster 3 peaks were associated with apoptosis, programmed cell death, and DNA damage; cluster 7 containing peaks found only in day 0 was linked with negative regulation of cell cycle control and the phenotypes of non-osseous mesenchyme-derived cells (Additional files 4 and 5). These functional annotations associated with Runx2 peaks are generally consistent with the progression of MC3T3-E1 differentiation, supporting a temporal transcription network programmed by Runx2.

### Runx2 binding patterns at osteogenic genes are predictive of potential Runx2 targets

**Figure 4:**
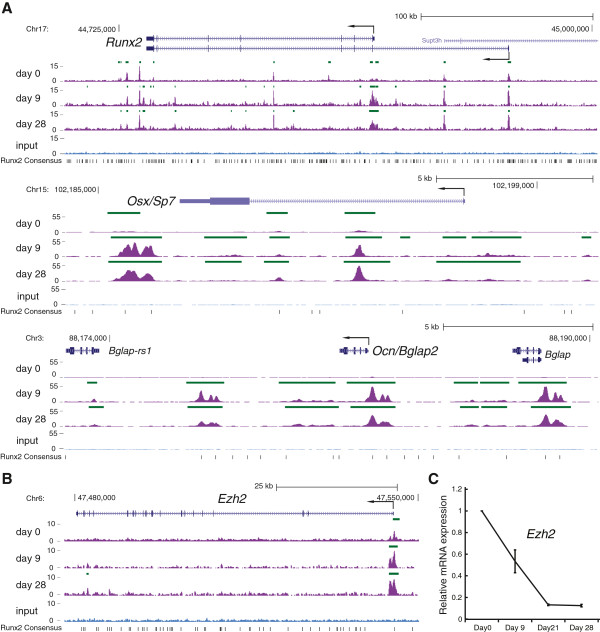
**Runx2 binding at known osteogenic genes and a potential target, *Ezh2***, **during osteoblastogenesis. (A)** Runx2 binding at three known osteogenic genes, *Runx2*, *Osx*, and *Ocn*, during three stages of osteogenesis: proliferation (day 0); matrix deposition (day 9); and mineralization (day 28). Gene annotation follows standard gene prediction display conventions used by the UCSC genome browser (exons, solid boxes; introns, solid lines; direction of gene transcription, arrows). Positions of Runx2 peaks called by MACS (green bars) and Runx2 consensus motif (TGTGGT; solid black bar) are also depicted. **(B)***Ezh2* locus bound by Runx2 with increasing Runx2 enrichment over temporal stages of osteogenesis. **(C)***Ezh2* expression during osteogenesis (relative to day 0 levels) were determined by quantitative RT-PCR and normalized by *Hprt1* mRNA levels. Relative mRNA levels are plotted as mean ± SEM from three independent biological replicates.

To discover previously unknown Runx2 target genes, we first determined the Runx2 binding patterns of well-known Runx2 target genes found in differentiation cluster 4; for example, *Runx2*, *Osx/Sp7*, and *Ocn/Bglap2* (Figure 4A; Additional file 6), and *Bsp/Ibsp* (Additional file 7). For these genes Runx2 binding was distributed in promoters, the gene body (introns/exons) and sites distal from the gene body. Furthermore, Runx2 enrichment increased in the loci of these genes during osteoblast differentiation at matrix and mineralization stages. We then examined the genes associated with cluster 1 peaks (Additional file 8), and identified genes, including *Ezh2* (enchancer of zeste homolog 2) (Figure 4B), with Runx2 binding profiles that displayed a ubiquitous but increasing level of Runx2 occupancy. Ezh2 is a component of PRC2 that epigenetically regulates gene expression by methylating histone H3 lysine 27 and was recently found to be involved in commitment of mesenchymal stem cells towards the osteoblast lineage [27]. The 5′ proximal promoter region of *Ezh2* is bound by Runx2 (Figure 4B; Additional file 6) and shows an increase in reads (occupancy) during differentiation. When *Ezh2* mRNA levels were measured during osteoblast differentiation, we found that the highest expression occurred in proliferating MC3T3-E1 cells, when the lowest amount of Runx2 binding was observed (Figure 4C). The striking decrease in *Ezh2* mRNA levels with increased Runx2 binding suggests that the transcription of *Ezh2* is potentially regulated by Runx2.

### Genes regulated by Runx2 exhibit distinct Runx2 binding profiles

**Figure 5:**
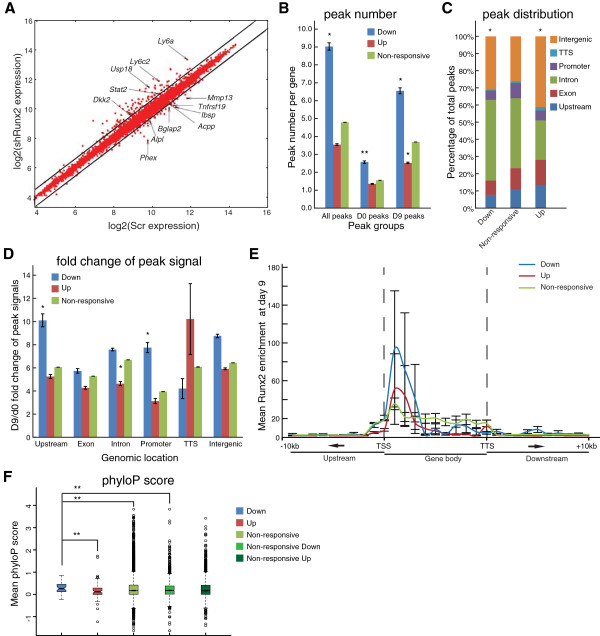
**Correlation of Runx2 occupancy and Runx2-reponsive genes identifies novel targets. (A)** Expression levels of genes responsive to Runx2 silencing in differentiated MC3T3-E1 cells. Values are plotted as the log2(expression level) from shRunx2-expressing cells (vertical axis) versus control shRNA expression (horizontal axis). Each point represents mean mRNA expression level from three independent biological replicates. Several representative genes are labeled. Diagonal lines demarcate the threshold for significant increase or decrease (≥1.5-fold) in expression. **(B-D)** Runx2 peaks associated with upregulated (Up), downregulated (Down), or unchanged (Non-responsive) gene expression upon Runx2 knockdown were compared by: average peak number per gene **(B)**, and peak distribution **(C)** and fold change of peak signals (d9 versus d0) across genomic locations **(D)** with shRunx2 non-responsive genes as a control. In **(B)**, three groups were compared to non-responsive genes: all peaks in shRunx2-regulated genes (All peaks), all peaks present at day 9 in shRunx2-regulated genes (D9 peaks), and all peaks present at day 0 in shRunx2 regulated genes (D0 peaks). Values are mean ± SEM **(B,D)** and statistical significance (*P < 0.01, **P < 0.05) determined by Mann-Whitney test **(B,D)** or Fisher’s exact test **(C)**. **(E)** Runx2 enrichment across gene bodies (±10 kb) of genes downregulated (Down), upregulated (Up), and unchanged (Non-responsive) by shRunx2 treatment at day 9. Mean signal ratios (IP/Input) at each genomic region were determined using PeaksToGenes. Error bars represent SEM. **(F)** Mean phyloP conservation scores of Runx2 motifs associated with genes significantly (fold change ≥1.5, FDR <0.05) downregulated (Down), upregulated (Up), or unchanged (Non-responsive) upon shRunx2-treatment. Conservation of Runx2 motifs was compared between shRunx2 downregulated and upregulated genes and length-matched non-responsive genes (Non-responsive, Down and Up, respectively) and statistical significance determined (**P < 0.05) by Kolmogorov-Smirnov test.

To characterize the Runx2 binding profiles in genes that are transcriptionally regulated by Runx2, we performed gene expression profiling at day 9 in MC3T3-E1 cells treated with control (Scr) short hairpin RNA (shRNA) or a Runx2-specific shRNA (shRunx2) that knocks down Runx2 expression. Runx2 protein levels decreased by 80% in cells treated with shRunx2 (Additional file 9). This knockdown in turn inhibited expression of differentiation marker genes and osteoblastogenesis as demonstrated by decreased Alp staining (Additional file 9). We found 159 genes whose expression was responsive to Runx2 knockdown, with |log_2_(I_shRunx2_/I_Scr_)| > 1.5, and a false discovery rate (FDR) <0.05, where I is the measured normalized probe-set intensity. These genes included 115 up- and 44 down-regulated genes (Figure 5A). The 15 genes most responsive to Runx2 knockdown (Table 1) included the well-defined Runx2 targets *Ocn/Bglap2*, *Bsp/Ibsp*, and *Mmp13*, which are known to be activated by Runx2 [5]. Notably, the genes most upregulated by shRunx2 have not been characterized as Runx2 targets (Table 1) except for *Usp18*, which encodes a protein involved in the ubiquitin degradation pathway [28].

**Table 1:** Top 15 genes upregulated or downregulated during shRunx2 treatment when compared to scramble shRNA

**Down**		**Up**	
**Symbol**	**Fold change**	**Symbol**	**Fold change**
*Phex*	0.26	I830012O16Rik	4.73
*H19*	0.36	*Ifi27l2a*	3.83
*Ibsp*	0.46	*Oas2*	3.78
*Mmp13*	0.46	D14Ertd668e	3.62
*Mid2*	0.49	*Ifit3*	3.37
*Edil3*	0.50	*Gm12250*	3.27
*Tnfrsf19*	0.53	*Ifit1*	3.25
*Bglap2*	0.56	*Ly6c1*	3.24
*C87414*	0.56	*Usp18*	3.09
*Gpr116*	0.56	BC023105	3.05
*Pdlim1*	0.57	*Iigp1*	3.05
*Hs3st3a1*	0.57	*Ddx60*	2.88
*Id3*	0.58	*Gm4951*	2.87
*Chac1*	0.58	*Ly6a*	2.87
*Il33*	0.58	*Ly6c2*	2.85

We characterized the relationship between Runx2 occupancy and genes affected by shRunx2 knockdown, compared to non-responsive genes. The number of peaks, genomic distribution of peaks, and fold change of peak signals were compared among the gene groups at days 0 and 9 (Figure 5B-D). Genes that were downregulated by shRunx2 at both days 0 and 9 had more Runx2 peaks when compared to the control non-responsive genes (Figure 5B). It should be noted, however, that shRunx2 downregulated genes are longer than the upregulated genes (Additional file 10). This finding was also observed when we included the Runx2 binding profiles at day 28 for Runx2-regulated genes (Additional file 10). In contrast, the shRunx2 upregulated genes that appeared on day 9 had fewer Runx2 peaks compared to control genes (Figure 5B). There were also significant differences in overall peak distribution between shRunx2 responsive genes and control (Figure 5C). Genes that were downregulated tended to exhibit more intronic and intergenic enrichment of Runx2 peaks, while shRunx2 upregulated genes were strongly enriched in intergenic but reduced in intronic binding. We further examined the distribution of Runx2 peaks in up- and down-regulated genes as a function of changes in Runx2 binding during differentiation from days 0 to 9 (Figure 5D). The fold change in day 9/day 0 peak signals showed increased Runx2 binding predominantly at upstream and promoter regions for the shRunx2 downregulated genes; in shRunx2 upregulated genes, Runx2 binding diminished in the introns and showed no significant change in promoter regions. Therefore, shRunx2 downregulated and upregulated genes exhibited distinct preferences for Runx2 binding in genomic loci as reflected by peak distributions in Figure 5B,C, and in relation to differentiation (Figure 5D).

To complement the above analysis examining the genome-wide distribution of Runx2 responsive peaks, we used the PeaksToGenes program [29] to determine the enrichment of day 9 Runx2 signals at defined intervals within and surrounding the gene bodies (Figure 5E). For shRunx2 downregulated genes, Runx2 binding had the strongest enrichment surrounding TSSs, which includes proximal promoter, 5′ UTR, exons and introns all within five contiguous deciles (Figure 5E; Additional file 11). This analysis demonstrated that, of genes affected by shRunx2, 66.1% of upregulated genes have a low level of Runx2 binding (cluster V in Figure S5B in Additional file 11) but most downregulated genes (69.1%) have higher level intensities of Runx2 binding (Figure S5A,B in Additional file 11). This finding shows that Runx2 responsive genes at day 9 (shRunx2/Scr) are primarily regulated by Runx2 surrounding TSSs.

As another computational analysis, EMBER (Expectation Maximization of Binding and Expression pRofiles) [30] was used to relate measured changes in gene expression to the spectrum of Runx2 occupancy observed during osteoblast differentiation (Figure 3B,C; Additional file 12). In analogy with discovering a sequence motif from a collection of functionally related DNA sequences, EMBER optimizes an ‘expression pattern’ from a collection of genes related by patterns of transcription factor binding and uses this motif to determine which genes are regulatory targets of the transcription factor (details of EMBER are summarized in Additional file 3). Using this approach, we discovered and compared expression patterns from different sets of Runx2 binding regions (Figure 3). The Runx2 peaks were partitioned into 42 subsets of Runx2 binding regions (7 clusters with 6 genomic location categories) and it was observed that all groups of Runx2 binding show a correlative relationship to gene expression during osteoblast differentiation (Additional file 12). We noted, however, that intronic Runx2 binding regions - for all time-dependent patterns of Runx2 occupancy - are less informative than other groups, indicating that intronic Runx2 binding may be less useful than binding at other class elements as a predictor of gene regulation.

Finally, downregulated and upregulated genes were compared on the basis of their evolutionary conservation (Figure 5F). Functional genomic elements are often characterized by conservation, which has been used to guide the prediction of transcription factor binding sites [31,32]. This finding has been shown to distinguish functionally verified from un-verified binding sites in a large-scale study of transcription factor binding site function on human promoters [33]. For each ChIP-Seq peak, a Runx motif was used to identify the single most likely Runx2 binding site and the mean phyloP score [34] (for conservation among 30 vertebrate species) across the binding sites was computed. For each gene, phyloP scores were averaged among all ChIP-Seq peaks that were associated with individual genes to give an average measure of Runx2 conservation. We found that genes downregulated by shRunx2 had Runx2 binding sequences that were more conserved than those in upregulated or non-responsive genes (Figure 5F), suggesting that Runx2 regulation may be more evolutionarily conserved in genes that are activated by Runx2 during osteoblastogenesis.

Taken together, the complementary methods described above suggest that Runx2 employs different mechanisms to regulate gene expression: 1) shRunx2 downregulated genes show increased Runx2 binding at promoter and far upstream regions; and 2) based on EMBER, intronic binding does not imply Runx2-mediated gene regulation to the same degree as Runx2 binding at promoter, exon and upstream regions. Thus, during the normal course of osteoblast differentiation, Runx2-activated genes (shRunx2 downregulated) are regulated through both promoter and non-promoter regions; and regulation of Runx2 repressed genes (shRunx2 upregulated) also occurs in promoter and other genomic regions, but with less Runx2 binding.

### Novel genes regulated by Runx2 through distinct regulatory elements

Among potential Runx2 targets, we identified *Tnfrsf19* (tumor necrosis factor receptor superfamily, member 19), which is involved in bone formation as a Wnt-responsive regulator of mesenchymal stem cell commitment to osteoblastic lineage [35]. *Tnfrsf19* exhibited enrichment of Runx2 binding to the promoter as well as intronic regions (Additional files 6 and 13). During differentiation of osteoblasts, *Tnfrsf19* mRNA levels increased dramatically more than 100-fold from day 0 to day 9 (Figure S7A in Additional file 13). Consistent with Affymetrix data, depletion of Runx2 resulted in significant decreases in *Tnfrsf19* expression, indicating direct Runx2 regulation (Figure S7B in Additional file 13). We found that Runx2 occupancy was increased at intronic and promoter regions of the *Tnsrsf19* locus during osteoblast differentiation (Figure S7C in Additional file 13).

**Figure 6:**
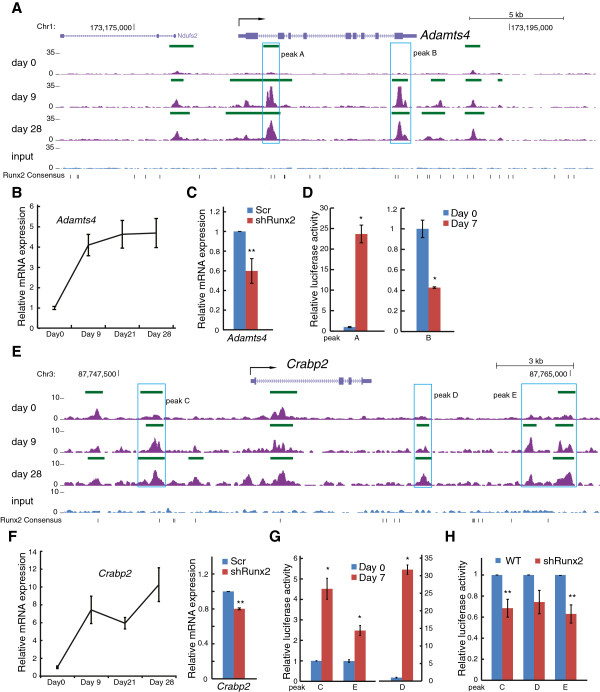
**Non-promoter association of Runx2 regulates novel targets *Adamts4 *and *Crabp2*. (A)** Increasing Runx2 enrichment was observed in the first intron (peak A, boxed region) and last exon (peak B, boxed region) of the *Adamts4* locus during osteogenic differentiation. **(B) ***Adamts4* mRNA levels (normalized to *Hprt1*) were significantly upregulated (*P* < 0.05) in differentiating MC3T3-E1 cells (days 9 to 28) when compared to proliferating cells (day 0). **(C)** Runx2 knockdown (shRunx2) decreases *Adamts4* expression, compared to a scrambled shRNA (Scr) control (**P < 0.05). **(D)** DNA sequences identical to peak regions A and B were cloned into individual pGL2-SV40-Luc reporters and relative luciferase activity was measured in transfected MC3T3-E1 cells and significant increases and decreases (*P < 0.01) in luciferase activity were observed for peak A and B reporters, respectively. **(E)** Increasing Runx2 enrichment was observed up- (peak C) and downstream (peaks D and E) of the *Crabp2* locus during osteogenic differentiation. **(F) ***Crabp2* expression increases during differentiation. Knockdown of Runx2 (by shRunx2) significantly reduces (**P < 0.05) the endogenous expression of *Crabp2* (right panel). **(G)** DNA sequences identical to peak regions C, D and E were cloned into individual pGL2-SV40-Luc reporters and relative luciferase activity was measured in transfected MC3T3-E1 cells at days 0 and 7 (*P < 0.01). **(H)** Runx2 knockdown by inducible shRNA results in a significant decrease (**P < 0.05) of luciferase activity mediated by peak C and E regions and a downward trend (*P* = 0.08) in luciferase activity regulated by peak D region. Statistical significance for all experiments was determined by Student’s *t*-test from mean ± SEM from three biological replicates.

*Adamts4* and *Crabp2* were selected for further analyses based on their responsiveness to shRunx2 (with fold change cutoff of 1.3; Additional file 14) and their Runx2 binding patterns predominantly in non-promoter regions. To test the functionality of Runx2 binding at these putative regulatory regions, we cloned non-promoter Runx2 binding regions and measured transcriptional activity by luciferase reporter assay. *Adamts4* is expressed in osteoblasts and osteocytes and encodes an enzyme that degrades aggrecan [36,37]. Runx2 exhibits multiple peaks across the *Adamts4* locus, with increased occupancy during osteoblast differentiation (Figure 6A; Additional file 6). *Adamts4* expression during osteoblast differentiation was increased at day 9 and remained steady to day 28 (Figure 6B). Knockdown of Runx2 suppressed the expression of *Adamts4* (Figure 6C) by 40%, supporting *Adamts4* as a direct target of Runx2 during osteoblastogenesis. We characterized the functional activity of two prominent Runx2 binding regions, peak A in intron 1 and peak B in the last exon (Figure 6A). The peak A region increased luciferase activity in MC3T3-E1 cells by over 20-fold. In contrast, peak B functioned as a suppressor of luciferase activity (Figure 6D). These results indicate that the two Runx2 sites can function as a positive and negative regulator of *Adamts4*; however, the weaker, negative regulation by peak B on the luciferase reporter may be due to the lack of a native chromatin context. The large increase in luciferase activity observed from peak A is consistent with a previous study that demonstrated Runx2 upregulates *ADAMTS4* in human SW1353 chondrosarcoma cells [38]. Here we established in osteoblasts that Runx2-mediated upregulation of *Adamts4* can occur at non-promoter regulatory elements, and is not restricted to the proximal promoter region as previously shown [38].

*Crabp2* is a cytoplasmic retinoic acid binding protein previously reported to be upregulated during osteoblastogenesis [39]. We observed that Runx2 constitutively occupies the *Crabp2* locus in the first intron, while binding increases upstream and downstream of the *Crabp2* gene body during differentiation (Figure 6E; Additional file 6). *Crabp2* was upregulated during MC3T3-E1 cell differentiation and knockdown of Runx2 decreased *Crabp2* mRNA level (Figure 6F). In proliferating cells, peak regions C, D and E demonstrated minimal luciferase reporter activity. However, in differentiating cells, all peaks exhibited a significant increase of luciferase activity, with peak region D showing a more than 30-fold activation (Figure 6G). The knockdown of Runx2 reduced luciferase activity (Figure 6H), further supporting the function of these regions in mediating Runx2 regulation of *Crabp2*.

These findings of novel genes bound and regulated by Runx2 through different types of genomic elements support an emerging concept that non-promoter elements can regulate gene transcription. Our results also indicate that Runx2 mediates complex fine-tuning of gene expression in osteoblasts by both activating and repressing regulatory elements that are located in non-promoter regions.

## Discussion

Through comprehensive genomic analysis of Runx2 by ChIP-Seq, we describe widespread Runx2 binding throughout the genome of differentiating osteoblasts. In addition to Runx2 interaction at promoters, we find Runx2 binding in non-promoter regions regulating novel targets that are silenced or expressed at different stages of osteoblast differentiation. Runx2 peaks cluster into temporal and functional categories associated with genes in a broad range of cellular programs, including bone development, negative regulation of proliferation, active matrix formation and mechanisms for mineral deposition that reflect the progression of osteogenesis. Our data have identified new Runx2-regulated genes (*Tnfrsf19*, *Adamts4*, *Crabp2*, and *Ezh2*) that have established roles in bone formation, and more importantly, have extended the understanding of Runx2-mediated gene regulation to a broader range of cellular functions during osteoblast differentiation.

### Runx2 binding patterns identify stage-dependent osteogenic programs

Time-dependent Runx2 binding patterns underlie the dynamic gene regulation by Runx2 during osteoblastogenesis. We identified a large number of peaks, consistent with the increasing protein levels of Runx2 from the early osteoprogenitor to the mature osteoblast/osteocyte. The results from binary clustering, together with subsequent GO term analyses by GREAT, identified many categories associated with skeletal development and bone homeostasis. These findings support our initial hypothesis that distinct binding patterns of Runx2 at different stages of osteoblastogenesis have novel functional implications.

Our clustering analysis partitioned Runx2 peaks into two main categories: less variable steady-state binding (cluster 1, ubiquitous peaks) and more dynamic binding groups (clusters 2 to 7, stage-specific clusters). Steady-state binding of Runx2 persists from proliferative pre-osteoblasts to differentiating osteoblasts with binding signals plateauing during matrix deposition and mineralization stages. Peaks in this category represent genes related to housekeeping processes such as protein folding, negative regulation of mitotic cell cycle, and mRNA catabolism and processes not previously related to Runx2 (Additional file 4). The GO terms associated with stage-specific clusters include negative regulators of other cell lineages (that is, fat and smooth muscle cells) as well as positive regulators of osteogenesis. Thus, dynamic Runx2 binding primes, enhances and stabilizes the osteoblast phenotype as well as suppresses non-osteoblast lineages. Many Runx2 bound genes in cluster 4 (days 9 & 28, differentiation) have been demonstrated to contribute to *in vivo* bone formation [40]. Thus, the genomic profiling of Runx2 binding in our *in vitro* model system is consistent with the known properties of Runx2 in bone formation. More importantly, our profiling reveals pathways previously unknown to be controlled by Runx2 underlying biological mechanisms of general cellular processes.

### Runx2 binding functions at both non-promoter and promoter regions

Only a small proportion of sequence-specific transcription factors, such as Myc, have narrow distributions of binding around proximal promoters of genes [41-44]. In contrast, non-promoter binding is recognized for other transcription factors, including STAT1, RUNX1, ERα, CTCF, and HNF4α [41,45-49]. In a previous study, overexpression of Runx2 in prostate cancer cells revealed extensive non-promoter binding [50]. In our study, endogenous Runx2 binding across the genome was characterized during osteoblastogenesis. We found that over 70% of Runx2 occupancy was localized to non-promoter regions (intergenic, intron, exon, TTS, and upstream regions that constitute the bulk of the genome) during the differentiation of osteoblasts. From days 0 to 9 there was a two-fold increase in the number of non-promoter peaks, indicating a functional association with the differentiation process. Runx2-dependent regulation through non-promoter peaks around the *Adamts4* and *Crabp2* genes provided direct evidence that non-promoter binding of Runx2 controls gene expression.

Although we have demonstrated the importance of non-promoter Runx2 binding events, Runx2 peaks at promoter regions have critical regulatory roles as well. In our data, Runx2 occupancy has the highest enrichment at promoter regions when compared with other genomic locations. Examples of this regulatory mode can be seen in some well-characterized Runx2 targets such as *Bsp* and *Ocn*, in line with established evidence that Runx2 can regulate these genes in a promoter-dependent manner [51-54]. The genes downregulated upon Runx2 silencing also displayed a clear enrichment of Runx2 signal at promoter regions (Figure 5B), exemplified in *Tnfrsf19*.

Gene expression in different biological settings is influenced by higher order three-dimensional chromatin complexes that involve looping of promoter and non-promoter elements, blurring the distinction of defined regulatory regions [55,56]. It is plausible that some Runx2 peaks in promoter and non-promoter regions may serve as nucleation sites for modifications of chromatin structures necessary for gene expression. Furthermore, transcription factors can interact with RNA polymerase II, CTCF, and other factors via higher-order chromatin conformations [55,57,58]. During osteoblastogenesis, Runx2 exhibited a distribution pattern among genomic elements similar to that of CTCF, suggesting that, like CTCF, Runx2 may have a functional role that extends beyond direct regulation of transcription. Consistent with this idea, Runx2 is able to form discernible foci associated with the nuclear matrix: a nuclear framework for organizing higher order chromatin structures. Runx2 truncated of the NMTS (nuclear matrix-targeting signal) domain results in diminished nuclear matrix association and disrupted expression of Runx2 target genes [59]. It is also noteworthy that the genes upregulated upon Runx2 knockdown have preferential Runx2 binding in intergenic regions, indicating that distal elements may have a regulatory role through long-range interactions. In addition, Runx2 binding at distal regions may contribute to chromatin remodeling through interacting with chromatin-modifying enzymes, as has been well documented at regulatory elements in osteoblasts and other cell models [12,13].

### The complexities of Runx2 binding and transcriptional regulation

Runx2 displays complex binding patterns similar to other lineage-specific transcription factors, such as PPAR-γ, MyoD, and GATA3 [16-19]. By systematic annotation of Runx2 peaks, multiple integrative analyses of gene expression combined with Runx2 binding profiles and direct experimental validation of individual targets, we defined Runx2 binding with biological outcomes during osteoblast differentiation. These analyses revealed that, for a small set of genes, the enrichment and binding patterns of Runx2 were indicators of gene expression. Genes that have decreased expression in the absence of Runx2 (by shRunx2 treatment) have a greater number of Runx2 peaks and greater fold change of peak signal at promoter and non-promoter regions when compared to non-responsive and gene length-matched controls. In contrast, genes upregulated by Runx2 knockdown tend to have fewer Runx2 peaks and smaller relative fold changes in peak signals when compared to controls. One notable finding that arose from our analysis was that genes that were downregulated in the absence of Runx2 had both a greater evolutionary conservation of Runx2 binding sites and tended to be longer than non-responsive and upregulated genes. It is unclear why this particular subset of genes (that is, genes normally activated by Runx2) would retain these features throughout evolution; however, this is an interesting point for future investigations. Although we demonstrated that Runx2 binding influences gene expression, a proportion of Runx2 peaks were found to have no direct function in transcriptional control of genes. This may be due to efficient but not complete knockdown of Runx2 by viral-mediated shRNA. Alternatively, similar to other transcription factors, many binding regions were found to be nonfunctional in transactivating luciferase reporters [33]. This finding suggests that binding of transcription factors may have a distinct function other than direct control of gene expression, consistent with previously described non-transcriptional functions and genome-organizing capabilities of Runx2 [59,60].

## Conclusions

Our findings provide a new level of understanding of the Runx2-mediated transcription program as defined by genome-wide Runx2 binding essential for osteoblastogenesis. Our data support that Runx2 functions at promoter and non-promoter regions at both previously known and novel targets. The impact of our study examining the global occupancy of endogenous Runx2 in differentiating osteoblasts sets a framework for novel mechanisms underlying bone biology.

## Materials and methods

### Cell culture

The calvaria-derived preosteoblast cell line MC3T3-E1 (Subclone 4) was obtained from ATCC (Manassas, VA, USA) and maintained in ascorbic acid-free alpha-MEM (Hyclone, Novato, CA, USA) supplemented with 10% fetal bovine serum (FBS; Hyclone), 2 mM L-glutamine, 100 U/ml of penicillin and 100 μg/ml streptomycin (Pen/Strep, Invitrogen, Carlsbad, CA,). To induce osteogenic differentiation, complete alpha-MEM was supplemented with 280 μM ascorbic acid and 10 mM beta-glycerophosphate (Sigma Aldrich, St. Louis, MO, USA). Cells were maintained at 37°C in a humidified 5% CO_2_ environment and media replaced every 2 to 3 days for the duration of all experiments.

### RNA isolation and quantitative PCR

Total RNA was isolated using Trizol reagent (Invitrogen) according to the manufacturers’ specifications. Total cellular RNA treated by DNaseI (Zymo, Irvine, CA, USA) was primed with random hexamers and reverse transcribed into cDNA using Superscript First-strand cDNA Synthesis Kit (Invitrogen) according to the manufacturer’s instructions. Gene expression was determined by quantitative real time PCR (qPCR) using iQ™ SYBR Green PCR Master Mix (BioRad, Hercules, CA, USA) in an ABI Prism 7300 thermocycler (Applied Biosystems, Foster City, CA, USA). For each gene, the expression level was normalized to that of *Hprt1* using 2^-ΔΔ*C*^_T_ method. Experiments were performed in triplicate and results are presented as mean values ± SEM. Primers for qPCR reactions were designed by FoxPrimer [61,62], and are available in Additional file 15.

### Runx2 knockdown and gene expression profiling

Lentiviruses carrying Runx2-shRNA and previously described control Scramble-shRNA [63] were used to infect MC3T3-E1 cells. Infected cells were subsequently detected by green fluorescent protein, sorted and grown to 90% confluency followed by osteogenic differentiation for 9 days. Knockdown experiments were performed in three biological replicates.

Microarray samples were handled following the manufacturers’ recommended protocols (Affymetrix, Santa Clara, CA, USA). Briefly, RNA isolated from MC3T3-E1 cells (day 0 and 9 scramble shRNA, and day 9 Runx2 shRNA) were reversely transcribed into cDNA using WT Expression Kit (Ambion, Austin, TX, USA), labeled and fragmented with GeneChip WT Terminal Labeling and Controls Kit. Labeled cDNAs were then hybridized to GeneChip Mouse Gene 1.0 ST Array rev.4 using a GeneChip Hybridization, Wash, and Stain Kit. Hybridization signals were obtained by GeneChip Scanner (Affymetrix). Microarray data were analyzed using Bioconductor (version 2.11) packages affy and limma in R (version 2.15.1) [64-67]. Briefly, after performing RMA (robust multichip average) normalization of microarray expression levels and filtering, differential expression was detected using a Bayesian moderated *t*-test. The Benjamini-Hochberg FDR [68] was applied to correct for multiple testing. The Affymetrix expression profiles were annotated to RefSeq genes [23]. These expression data have been deposited in the Gene Expresion Omnibus (GEO) database under accession number GSE53982.

### Western blot

Nuclei extracts were prepared from MC3T3-E1 cells using a protocol modified from Dignam *et al*. [69]. Primary antibodies and dilutions were: mouse anti-Runx2 monoclonal (Clone 8G5, MBL International, Woburn, MA, USA; 1:1,000); rabbit anti-H3 3H1 monoclonal (Cell Signaling, Danvers, MA, USA; 1:2,000). HRP-conjugated secondary antibodies and dilutions were: goat anti-mouse IgG (Santa Cruz, Dallas, TX, USA; 1:3,000); goat anti-rabbit IgG (Santa Cruz; 1:3,000). Detection of HRP was performed using a Western Lightning Plus Kit (Perkin Elmer, Waltham, MA, USA) followed by exposure onto Biomax Light film (Kodak, Rochester, NY, USA).

### Chromatin immunoprecipitation and high-throughput sequencing

At days 0, 9, and 28 of differentiation, approximately 1 × 10^8^ MC3T3-E1 cells were washed with PBS (phosphate-buffered saline) and then fixed on a plate with 1% formaldehyde for 8 minutes to crosslink DNA-protein complexes. The fixed cells were washed with ice-cold PBS, harvested, and pelleted. Nuclei extraction was performed using a protocol modified from Dignam *et al*. [69]. Isolated nuclei were sonicated using a Misonix S-4000 ultrasonic sonicator to obtain sheared chromatin ranging from 0.2 kb to 0.6 kb. Sheared chromatin was used for immunoprecipitation with Runx2 antibody (M-70, Santa Cruz) [70] or immunoglobulin G (IgG) (12-370, Millipore, Billerica, MA, USA) followed by purification using Protein-G Dynabeads (Invitrogen). Precipitated chromatin was washed with solutions of increasing salt concentration, and eluted and subsequently uncrosslinked at 65°C. DNA was recovered by phenol/chloroform/isoamyl alcohol extraction followed by ethanol precipitation. Libraries of purified DNA were generated using Illumina SR adapters (Illumina, San Diego, CA, USA) following the manufacturer’s manual. DNA libraries were selected for inserted fragments of 200 ± 50 bp, and single-end 36 base reads were generated on an Illumina Genome Analyzer II at the UMASS Deep-Seq core facility. Base calls and sequence reads were generated by Illumina CASAVA software (version 1.6; Illumina). Two independent biological repeats of Runx2 ChIP-Seq libraries were prepared for each time point, and two Input libraries were prepared with sonicated DNA from day 9 MC3T3-E1 cells.

### Analysis of ChIP-Seq data

Single-end 36 base sequences from Runx2 ChIP-Seq and input libraries were mapped to the mouse genome (assembly mm9) using Bowtie (version 0.12.8) [71]. Runx2 peaks and read counts were determined by MACS (version 1.4.1) [22] using default settings. Runx2 peaks that were significant at the *P* < 10^-10^ level were retained for subsequent analysis. For each of the three time points in our data, read counts were normalized to 10 million reads. The UCSC genome browser [72] was used to visualize Runx2 peaks.

Runx2 binding regions were classified on the basis of genomic location categories and annotated to known RefSeq genes [23]. Runx2 peaks were grouped into seven clusters based on the presence or absence of a peak. Runx2 peaks in each cluster were analyzed for GO terms using GREAT (version 2.0.2), using default association rules between ChIP-Seq peaks and annotated genes [73]. The conservation of Runx2 motifs associated with the genes responsive to shRunx2 were determined using phyloP [34]. Statistical significance was determined using Kolmogorov-Smirnov test.

PeaksToGenes [29,62] (Additional file 3) was used to test Runx2 binding in relation to the genes from microarray analysis. PeaksToGenes defines genomic intervals relative to all RefSeq genes, and in each window uses a non-parametric Wilcoxon rank sum test to calculate the probability and binding frequency. The comparisons were individually made between each responsive group and the non-responsive group to *Runx2* shRNA. Runx2 binding and Runx2-mediated transcription control were also evaluated by EMBER [30]. Analogous to discovering a sequence motif from functionally related DNA sequences [24], EMBER optimizes an expression pattern from a collection of genes’ expression data related by profiles of transcription factor binding and uses this information to determine which genes are potential regulatory targets of the transcription factor. For a detailed description of the PeaksToGenes and EMBER analysis, please refer to Additional file 3.

Functional genomic elements can be characterized by evolutionary conservation and have been shown to distinguish functionally verified from unverified transcription factor binding sites on human promoters [33]. For each ChIP-Seq peak (combined from day 0, 9, and 28 datasets), a Runx motif was used to identify the single most likely Runx2 binding site and the mean phyloP score [34] (for conservation among 30 vertebrate species) across the binding sites was computed. In order to compare conservation among genes that fell into different regulatory groups (shRunx2 downregulated, upregulated and non-responsive genes) based on our expression microarray measurements, phyloP scores were averaged among all ChIP-Seq peaks that were associated with individual genes to give an average measure of Runx2 conservation for each gene. To minimize the effect of gene length on Runx2 binding analysis, we compared the conservation of shRunx2-downregulated and -upregulated genes to randomly sampled, length-matched non-responsive genes. Statistical significance was determined by pair-wise conservation comparisons using Kolmogorov-Smirnov test.

The raw sequences and peak-related files in BED and WIG formats representing processed data have been deposited in the GEO database under accession number GSE54013.

### Luciferase assays and plasmid reporters

Selected Runx2 peak regions were cloned with MluI combined with BglII or XhoI into a pGL2-SV40-Luc reporter (Promega, Fitchburg, WI, USA). Primers used in cloning are listed in Additional file 16. A pGL2-SV40-Luc reporter with minimum SV40 promoter was used as mock control. Reporter plasmids with Runx2 peak regions and pGL2-SV40-Luc empty vector were co-transfected with pcDNA3.1-Runx2-WT or pcDNA3.1-EV into MC3T3 cells. Transfected cells were then differentiated for 7 days before luciferase activities were determined by Dual-Glo Luciferase Assay Kit (Promega). A second set of luciferase assays was done in differentiating MC3T3-E1 cells stably infected with a doxycycline-inducible pLKO-puro-Tet-on-Rx2shRNA lentiviral vector. This vector was constructed by re-cloning a previously validated Runx2 shRNA sequence [63] to Tet-pLKO-puro plasmid (catalog number 21915, Addgene, Cambridge, MA, USA). Luciferase activities from MC3T3-E1 cells treated with or without 2.5 μg/ml doxycycline were examined at day 7 after differentiation (Figure 6).

## Abbreviations

bp: base pair; ChIP: chromatin immunoprecipitation; FDR: false discovery rate; GEO: Gene Expresion Omnibus; GO: Gene Ontology; qPCR: quantitative real time polymerase chain reaction; shRNA: short hairpin RNA; TSS: transcription start site; TTS: transcription termination site; UTR: untranslated region, SEM, standard error of mean.

## Competing interests

The authors declare that they have no competing interests.

## Authors’ contributions

HW and JARG conceived and designed the experiments. HW performed the experiments. TWW, JRD, and HW performed bioinformatics data analysis. HW, JARG, TWW, and JRD interpreted the data with insights from PWLT, JBL, JLS, AVW, and GSS. HW, TWW, JARG, JRD, PWLT, JBL, and JLS wrote the paper with help from AVW and GSS. All authors read and approved the final manuscript.

## Supplementary Material

Additional file 1: Table S3 Summary of MC3T3 Runx2 ChIP-Seq. This table lists the read numbers and genome coverage of Runx2 ChIP-Seq libraries. Click here for file

Additional file 2: Table S4 Distribution patterns of Runx2 peaks across genomic locations. This table is related to Figure 2. Click here for file

Additional file 3 Detailed description of ChIP-PCR, ChIP-Seq with bioinformatics analysis, and supplemental figure legends. Click here for file

Additional file 4: Table S5 GREAT Gene Ontology terms. This table contains the top GO terms assigned by GREAT to clusters 1 to 7 defined in Figure 3A. Click here for file

Additional file 5: Figure S1 GO term analysis from GREAT for clusters 1 to 3 and 5 to 7 in Figure 3A. This is related to Figure 3. Click here for file

Additional file 6: Figure S8 Validation of Runx2 peaks by ChIP-PCR. This figure is related to Figures 4, 5 and 6. Click here for file

Additional file 7: Figure S2 Runx2 peaks associated with *Bsp* gene during differentiation. This figure is related to Figure 4. Click here for file

Additional file 8: Table S6 Detailed annotation of Runx2 peaks. This table is a meta-spreadsheet of Runx2 peaks annotated to RefSeq genes. Click here for file

Additional file 9: Figure S3 Validation of Runx2 knockdown in MC3T3 cells. This figure is related to Figure 5. Click here for file

Additional file 10: Figure S4 Additional characteristics of Runx2 binding in shRunx2 responsive genes. This figure is related to Figure 5. Click here for file

Additional file 11: Figure S5 PeaksToGenes analysis of Runx2 occupancy in *Runx2* shRNA-responsive genes. This figure is related to Figure 5E. Click here for file

Additional file 12: Figure S6 EMBER analyses of Runx2 binding in the genes differentially regulated by Runx2 knockdown. This figure is related to Figure 5. Click here for file

Additional file 13: Figure S7 Validation of novel Runx2 target *Tnfrsf19*. This figure is related to Figure 6. Click here for file

Additional file 14: Table S7 Genes responsive to *Runx2* shRNA. This table lists the genes that are up- or down- regulated by shRunx2 treatment in MC3T3 cells differentiated for 9 days. Click here for file

Additional file 15: Table S1 qPCR and ChIP-PCR primers. Click here for file

Additional file 16: Table S2 Cloning primers. This table contains the primers used for plasmid construction. Click here for file

## References

[B1] Komori T Signaling networks in RUNX2-dependent bone development J Cell Biochem 2011 112 750 755 10.1002/jcb.22994 21328448

[B2] Lian JB Stein GS Javed A van Wijnen AJ Stein JL Montecino M Hassan MQ Gaur T Lengner CJ Young DW Networks and hubs for the transcriptional control of osteoblastogenesis Rev Endocr Metab Disord 2006 7 1 16 17051438 10.1007/s11154-006-9001-5

[B3] Long F Building strong bones: molecular regulation of the osteoblast lineage Nat Rev Mol Cell Biol 2012 13 27 38 22189423 10.1038/nrm3254

[B4] Otto F Kanegane H Mundlos S Mutations in the RUNX2 gene in patients with cleidocranial dysplasia Hum Mutat 2002 19 209 216 10.1002/humu.10043 11857736

[B5] Hecht J Seitz V Urban M Wagner F Robinson PN Stiege A Dieterich C Kornak U Wilkening U Brieske N Zwingman C Kidess A Stricker S Mundlos S Detection of novel skeletogenesis target genes by comprehensive analysis of a Runx2(-/-) mouse model Gene Expr Patterns 2007 7 102 112 10.1016/j.modgep.2006.05.014 16829211

[B6] Vaes BL Ducy P Sijbers AM Hendriks JM van Someren EP de Jong NG van den Heuvel ER Olijve W van Zoelen EJ Dechering KJ Microarray analysis on Runx2-deficient mouse embryos reveals novel Runx2 functions and target genes during intramembranous and endochondral bone formation Bone 2006 39 724 738 10.1016/j.bone.2006.04.024 16774856

[B7] Jeon MJ Kim JA Kwon SH Kim SW Park KS Park SW Kim SY Shin CS Activation of peroxisome proliferator-activated receptor-gamma inhibits the Runx2-mediated transcription of osteocalcin in osteoblasts J Biol Chem 2003 278 23270 23277 10.1074/jbc.M211610200 12704187

[B8] Zhang YY Li X Qian SW Guo L Huang HY He Q Liu Y Ma CG Tang QQ Down-regulation of type I Runx2 mediated by dexamethasone is required for 3T3-L1 adipogenesis Mol Endocrinol 2012 26 798 808 10.1210/me.2011-1287 22422618 PMC5417096

[B9] Gersbach CA Byers BA Pavlath GK Garcia AJ Runx2/Cbfa1 stimulates transdifferentiation of primary skeletal myoblasts into a mineralizing osteoblastic phenotype Exp Cell Res 2004 300 406 417 10.1016/j.yexcr.2004.07.031 15475005

[B10] Schroeder TM Jensen ED Westendorf JJ Runx2: a master organizer of gene transcription in developing and maturing osteoblasts Birth Defects Res C Embryo Today 2005 75 213 225 10.1002/bdrc.20043 16187316

[B11] Zaidi SK Young DW Montecino MA Lian JB van Wijnen AJ Stein JL Stein GS Mitotic bookmarking of genes: a novel dimension to epigenetic control Nat Rev Genet 2010 11 583 589 10.1038/nrg2827 20628351 PMC3033599

[B12] Bradley EW McGee-Lawrence ME Westendorf JJ Hdac-mediated control of endochondral and intramembranous ossification Crit Rev Eukaryot Gene Expr 2011 21 101 113 10.1615/CritRevEukarGeneExpr.v21.i2.10 22077150 PMC3218555

[B13] Pelletier N Champagne N Stifani S Yang XJ MOZ and MORF histone acetyltransferases interact with the Runt-domain transcription factor Runx2 Oncogene 2002 21 2729 2740 10.1038/sj.onc.1205367 11965546

[B14] Villagra A Cruzat F Carvallo L Paredes R Olate J van Wijnen AJ Stein GS Lian JB Stein JL Imbalzano AN Montecino M Chromatin remodeling and transcriptional activity of the bone-specific osteocalcin gene require CCAAT/enhancer-binding protein beta-dependent recruitment of SWI/SNF activity J Biol Chem 2006 281 22695 22706 10.1074/jbc.M511640200 16772287

[B15] van Wijnen AJ Stein GS Gergen JP Groner Y Hiebert SW Ito Y Liu P Neil JC Ohki M Speck N Nomenclature for Runt-related (RUNX) proteins Oncogene 2004 23 4209 4210 10.1038/sj.onc.1207758 15156174

[B16] Mikkelsen TS Xu Z Zhang X Wang L Gimble JM Lander ES Rosen ED Comparative epigenomic analysis of murine and human adipogenesis Cell 2010 143 156 169 10.1016/j.cell.2010.09.006 20887899 PMC2950833

[B17] Cao Y Yao Z Sarkar D Lawrence M Sanchez GJ Parker MH MacQuarrie KL Davison J Morgan MT Ruzzo WL Gentleman RC Tapscott SJ Genome-wide MyoD binding in skeletal muscle cells: a potential for broad cellular reprogramming Dev Cell 2010 18 662 674 10.1016/j.devcel.2010.02.014 20412780 PMC2910615

[B18] Zhang JA Mortazavi A Williams BA Wold BJ Rothenberg EV Dynamic transformations of genome-wide epigenetic marking and transcriptional control establish T cell identity Cell 2012 149 467 482 10.1016/j.cell.2012.01.056 22500808 PMC3336965

[B19] Handoko L Xu H Li G Ngan CY Chew E Schnapp M Lee CW Ye C Ping JL Mulawadi F Wong E Sheng J Zhang Y Poh T Chan CS Kunarso G Shahab A Bourque G Cacheux-Rataboul V Sung WK Ruan Y Wei CL CTCF-mediated functional chromatin interactome in pluripotent cells Nat Genet 2011 43 630 638 10.1038/ng.857 21685913 PMC3436933

[B20] Wang D Christensen K Chawla K Xiao G Krebsbach PH Franceschi RT Isolation and characterization of MC3T3-E1 preosteoblast subclones with distinct in vitro and in vivo differentiation/mineralization potential J Bone Miner Res 1999 14 893 903 10.1359/jbmr.1999.14.6.893 10352097

[B21] Choi JY Lee BH Song KB Park RW Kim IS Sohn KY Jo JS Ryoo HM Expression patterns of bone-related proteins during osteoblastic differentiation in MC3T3-E1 cells J Cell Biochem 1996 61 609 618 10.1002/(SICI)1097-4644(19960616)61:4<609::AID-JCB15>3.0.CO;2-A 8806085

[B22] Zhang Y Liu T Meyer CA Eeckhoute J Johnson DS Bernstein BE Nusbaum C Myers RM Brown M Li W Liu XS Model-based analysis of ChIP-Seq (MACS) Genome Biol 2008 9 R137 10.1186/gb-2008-9-9-r137 18798982 PMC2592715

[B23] Pruitt KD Tatusova T Maglott DR NCBI reference sequences (RefSeq): a curated non-redundant sequence database of genomes, transcripts and proteins Nucleic Acids Res 2007 35 D61 D65 10.1093/nar/gkl842 17130148 PMC1716718

[B24] Bailey TL Boden M Buske FA Frith M Grant CE Clementi L Ren J Li WW Noble WS MEME SUITE: tools for motif discovery and searching Nucleic Acids Res 2009 37 W202 W208 10.1093/nar/gkp335 19458158 PMC2703892

[B25] Lee BK Iyer VR Genome-wide studies of CCCTC-binding factor (CTCF) and cohesin provide insight into chromatin structure and regulation J Biol Chem 2012 287 30906 30913 10.1074/jbc.R111.324962 22952237 PMC3438923

[B26] Portales-Casamar E Thongjuea S Kwon AT Arenillas D Zhao X Valen E Yusuf D Lenhard B Wasserman WW Sandelin A JASPAR 2010: the greatly expanded open-access database of transcription factor binding profiles Nucleic Acids Res 2010 38 D105 D110 10.1093/nar/gkp950 19906716 PMC2808906

[B27] Wei Y Chen YH Li LY Lang J Yeh SP Shi B Yang CC Yang JY Lin CY Lai CC Hung MC CDK1-dependent phosphorylation of EZH2 suppresses methylation of H3K27 and promotes osteogenic differentiation of human mesenchymal stem cells Nat Cell Biol 2011 13 87 94 10.1038/ncb2139 21131960 PMC3076036

[B28] Malhotra S Morcillo-Suarez C Nurtdinov R Rio J Sarro E Moreno M Castillo J Navarro A Montalban X Comabella M Roles of the ubiquitin peptidase USP18 in multiple sclerosis and the response to interferon-beta treatment Eur J Neurol 2013 20 1390 1397 10.1111/ene.12193 23700969

[B29] PeaksToGenes [https://github.com/peakstogenes/PeaksToGenes]

[B30] Maienschein-Cline M Zhou J White KP Sciammas R Dinner AR Discovering transcription factor regulatory targets using gene expression and binding data Bioinformatics 2012 28 206 213 10.1093/bioinformatics/btr628 22084256 PMC3259433

[B31] Kellis M Patterson N Endrizzi M Birren B Lander ES Sequencing and comparison of yeast species to identify genes and regulatory elements Nature 2003 423 241 254 10.1038/nature01644 12748633

[B32] Stark A Lin MF Kheradpour P Pedersen JS Parts L Carlson JW Crosby MA Rasmussen MD Roy S Deoras AN Ruby JG Brennecke J Hodges E Hinrichs AS Caspi A Paten B Park SW Han MV Maeder ML Polansky BJ Robson BE Aerts S van Helden J Hassan B Gilbert DG Eastman DA Rice M Weir M Hahn MW Park Y Discovery of functional elements in 12 Drosophila genomes using evolutionary signatures Nature 2007 450 219 232 10.1038/nature06340 17994088 PMC2474711

[B33] Whitfield TW Wang J Collins PJ Partridge EC Aldred SF Trinklein ND Myers RM Weng Z Functional analysis of transcription factor binding sites in human promoters Genome Biol 2012 13 R50 10.1186/gb-2012-13-9-r50 22951020 PMC3491394

[B34] Siepel A Pollard KS Haussler D Apostolico A, Guerra C, Istrail S, Pevzner P, Waterman M New methods for detecting lineage-specific selection Research in Computational Molecular Biology, 10th Annual International Conference, RECOMB 2006; April 2-5, 2006: Venice, Italy 2006 Berlin: Springer-Verlag 190 205

[B35] Qiu W Hu Y Andersen TE Jafari A Li N Chen W Kassem M Tumor necrosis factor receptor superfamily member 19 (TNFRSF19) regulates differentiation fate of human mesenchymal (stromal) stem cells through canonical Wnt signaling and C/EBP J Biol Chem 2010 285 14438 14449 10.1074/jbc.M109.052001 20223822 PMC2863178

[B36] Sone S Nakamura M Maruya Y Takahashi I Mizoguchi I Mayanagi H Sasano Y Expression of versican and ADAMTS during rat tooth eruption J Mol Histol 2005 36 281 288 10.1007/s10735-005-5534-2 16200461

[B37] Wang K Vishwanath P Eichler GS Al-Sebaei MO Edgar CM Einhorn TA Smith TF Gerstenfeld LC Analysis of fracture healing by large-scale transcriptional profile identified temporal relationships between metalloproteinase and ADAMTS mRNA expression Matrix Biol 2006 25 271 281 10.1016/j.matbio.2006.02.001 16584876

[B38] Thirunavukkarasu K Pei Y Moore TL Wang H Yu XP Geiser AG Chandrasekhar S Regulation of the human ADAMTS-4 promoter by transcription factors and cytokines Biochem Biophys Res Commun 2006 345 197 204 10.1016/j.bbrc.2006.04.023 16677612

[B39] Beck GR Jr Zerler B Moran E Gene array analysis of osteoblast differentiation Cell Growth Differ 2001 12 61 83 11243467

[B40] Baldridge D Shchelochkov O Kelley B Lee B Signaling pathways in human skeletal dysplasias Annu Rev Genomics Hum Genet 2010 11 189 217 10.1146/annurev-genom-082908-150158 20690819

[B41] Lee BK Bhinge AA Battenhouse A McDaniell RM Liu Z Song L Ni Y Birney E Lieb JD Furey TS Crawford GE Iyer VR Cell-type specific and combinatorial usage of diverse transcription factors revealed by genome-wide binding studies in multiple human cells Genome Res 2012 22 9 24 10.1101/gr.127597.111 22090374 PMC3246210

[B42] Birney E Stamatoyannopoulos J Dutta A Guigó R Gingeras TR Margulies EH Weng Z Snyder M Dermitzakis ET Thurman RE Kuehn MS Taylor CM Neph S Koch CM Asthana S Malhotra A Adzhubei I Greenbaum J Andrews RM Flicek P Boyle PJ Cao H Carter NP Clelland GK Davis S Day N Dhami P Dillon SC Dorschner MO Fiegler H Identification and analysis of functional elements in 1% of the human genome by the ENCODE pilot project Nature 2007 447 799 816 10.1038/nature05874 17571346 PMC2212820

[B43] Neph S Vierstra J Stergachis AB Reynolds AP Haugen E Vernot B Thurman RE John S Sandstrom R Johnson AK Maurano MT Humbert R Rynes E Wang H Vong S Lee K Bates D Diegel M Roach V Dunn D Neri J Schafer A Hansen RS Kutyavin T Giste E Weaver M Canfield T Sabo P Zhang M Balasundaram G An expansive human regulatory lexicon encoded in transcription factor footprints Nature 2012 489 83 90 10.1038/nature11212 22955618 PMC3736582

[B44] Farnham PJ Insights from genomic profiling of transcription factors Nat Rev Genet 2009 10 605 616 10.1038/nrg2636 19668247 PMC2846386

[B45] Robertson G Hirst M Bainbridge M Bilenky M Zhao Y Zeng T Euskirchen G Bernier B Varhol R Delaney A Thiessen N Griffith OL He A Marra M Snyder M Jones S Genome-wide profiles of STAT1 DNA association using chromatin immunoprecipitation and massively parallel sequencing Nat Methods 2007 4 651 657 10.1038/nmeth1068 17558387

[B46] Stender JD Kim K Charn TH Komm B Chang KC Kraus WL Benner C Glass CK Katzenellenbogen BS Genome-wide analysis of estrogen receptor alpha DNA binding and tethering mechanisms identifies Runx1 as a novel tethering factor in receptor-mediated transcriptional activation Mol Cell Biol 2010 30 3943 3955 10.1128/MCB.00118-10 20547749 PMC2916448

[B47] Tijssen MR Cvejic A Joshi A Hannah RL Ferreira R Forrai A Bellissimo DC Oram SH Smethurst PA Wilson NK Wang X Ottersbach K Stemple DL Green AR Ouwehand WH Gottgens B Genome-wide analysis of simultaneous GATA1/2, RUNX1, FLI1, and SCL binding in megakaryocytes identifies hematopoietic regulators Dev Cell 2011 20 597 609 10.1016/j.devcel.2011.04.008 21571218 PMC3145975

[B48] Visel A Blow MJ Li Z Zhang T Akiyama JA Holt A Plajzer-Frick I Shoukry M Wright C Chen F Afzal V Ren B Rubin EM Pennacchio LA ChIP-seq accurately predicts tissue-specific activity of enhancers Nature 2009 457 854 858 10.1038/nature07730 19212405 PMC2745234

[B49] Weltmeier F Borlak J A high resolution genome-wide scan of HNF4alpha recognition sites infers a regulatory gene network in colon cancer PLoS One 2011 6 e21667 10.1371/journal.pone.0021667 21829439 PMC3145629

[B50] Little GH Noushmehr H Baniwal SK Berman BP Coetzee GA Frenkel B Genome-wide Runx2 occupancy in prostate cancer cells suggests a role in regulating secretion Nucleic Acids Res 2012 40 3538 3547 10.1093/nar/gkr1219 22187159 PMC3333873

[B51] Javed A Barnes GL Jasanya BO Stein JL Gerstenfeld L Lian JB Stein GS: runt homology domain transcription factors (Runx, Cbfa, and AML) mediate repression of the bone sialoprotein promoter: evidence for promoter context-dependent activity of Cbfa proteins Mol Cell Biol 2001 21 2891 2905 10.1128/MCB.21.8.2891-2905.2001 11283267 PMC86918

[B52] Paredes R Arriagada G Cruzat F Villagra A Olate J Zaidi K van Wijnen A Lian JB Stein GS Stein JL Montecino M Bone-specific transcription factor Runx2 interacts with the 1alpha,25-dihydroxyvitamin D3 receptor to up-regulate rat osteocalcin gene expression in osteoblastic cells Mol Cell Biol 2004 24 8847 8861 10.1128/MCB.24.20.8847-8861.2004 15456860 PMC517904

[B53] Paz J Wade K Kiyoshima T Sodek J Tang J Tu Q Yamauchi M Chen J Tissue- and bone cell-specific expression of bone sialoprotein is directed by a 9.0 kb promoter in transgenic mice Matrix Biol 2005 24 341 352 10.1016/j.matbio.2005.05.009 15970437

[B54] Roca H Phimphilai M Gopalakrishnan R Xiao G Franceschi RT Cooperative interactions between RUNX2 and homeodomain protein-binding sites are critical for the osteoblast-specific expression of the bone sialoprotein gene J Biol Chem 2005 280 30845 30855 10.1074/jbc.M503942200 16000302

[B55] Li G Ruan X Auerbach RK Sandhu KS Zheng M Wang P Poh HM Goh Y Lim J Zhang J Sim HS Peh SQ Mulawadi FH Ong CT Orlov YL Hong S Zhang Z Landt S Raha D Euskirchen G Wei CL Ge W Wang H Davis C Fisher-Aylor KI Mortazavi A Gerstein M Gingeras T Wold B Sun Y Extensive promoter-centered chromatin interactions provide a topological basis for transcription regulation Cell 2012 148 84 98 10.1016/j.cell.2011.12.014 22265404 PMC3339270

[B56] Kowalczyk MS Hughes JR Garrick D Lynch MD Sharpe JA Sloane-Stanley JA McGowan SJ De Gobbi M Hosseini M Vernimmen D Brown JM Gray NE Collavin L Gibbons RJ Flint J Taylor S Buckle VJ Milne TA Wood WG Higgs DR Intragenic enhancers act as alternative promoters Mol Cell 2012 45 447 458 10.1016/j.molcel.2011.12.021 22264824

[B57] Lieberman-Aiden E van Berkum NL Williams L Imakaev M Ragoczy T Telling A Amit I Lajoie BR Sabo PJ Dorschner MO Sandstrom R Bernstein B Bender MA Groudine M Gnirke A Stamatoyannopoulos J Mirny LA Lander ES Dekker J Comprehensive mapping of long-range interactions reveals folding principles of the human genome Science 2009 326 289 293 10.1126/science.1181369 19815776 PMC2858594

[B58] Tan-Wong SM Zaugg JB Camblong J Xu Z Zhang DW Mischo HE Ansari AZ Luscombe NM Steinmetz LM Proudfoot NJ Gene loops enhance transcriptional directionality Science 2012 338 671 675 10.1126/science.1224350 23019609 PMC3563069

[B59] Zaidi SK Javed A Choi JY van Wijnen AJ Stein JL Lian JB Stein GS A specific targeting signal directs Runx2/Cbfa1 to subnuclear domains and contributes to transactivation of the osteocalcin gene J Cell Sci 2001 114 3093 3102 11590236 10.1242/jcs.114.17.3093

[B60] Young DW Hassan MQ Pratap J Galindo M Zaidi SK Lee SH Yang X Xie R Javed A Underwood JM Furcinitti P Imbalzano AN Penman S Nickerson JA Montecino MA Lian JB Stein JL van Wijnen AJ Stein GS Mitotic occupancy and lineage-specific transcriptional control of rRNA genes by Runx2 Nature 2007 445 442 446 10.1038/nature05473 17251981

[B61] FoxPrimer qPCR Primer Design Suite [http://www.foxprimer.org]

[B62] Dobson JR Nuclear organization in breast cance PhD thesis 2013 UMASS Medical School

[B63] Pratap J Wixted JJ Gaur T Zaidi SK Dobson J Gokul KD Hussain S van Wijnen AJ Stein JL Stein GS Lian JB Runx2 transcriptional activation of Indian Hedgehog and a downstream bone metastatic pathway in breast cancer cells Cancer Res 2008 68 7795 7802 10.1158/0008-5472.CAN-08-1078 18829534 PMC2596479

[B64] R: A Language and Environment for Statistical Computing [http://www.R-project.org]

[B65] Gautier L Cope L Bolstad BM Irizarry RA affy–analysis of Affymetrix GeneChip data at the probe level Bioinformatics 2004 20 307 315 10.1093/bioinformatics/btg405 14960456

[B66] Gentleman RC Carey VJ Bates DM Bolstad B Dettling M Dudoit S Ellis B Gautier L Ge Y Gentry J Hornik K Hothorn T Huber W Iacus S Irizarry R Leisch F Li C Maechler M Rossini AJ Sawitzki G Smith C Smyth G Tierney L Yang JY Zhang J Bioconductor: open software development for computational biology and bioinformatics Genome Biol 2004 5 R80 10.1186/gb-2004-5-10-r80 15461798 PMC545600

[B67] Smyth GK Gentleman R, Carey VJ, Huber W, Irizarry RA, Dudoit S Limma: linear models for microarray data Bioinformatics and Computational Biology Solutions Using R and Bioconductor 2005 New York: Springer 397 420 [Gail M, Krickeberg K, Samet J, Tsiatis A, Wong W (Series Editors): *Statistics for Biology and Health*]

[B68] Benjamini Y Hochberg Y Controlling the false discovery rate - a practical and powerful approach to multiple testing J R Stat Soc Series B Methodol 1995 57 289 300

[B69] Dignam JD Lebovitz RM Roeder RG Accurate transcription initiation by RNA polymerase II in a soluble extract from isolated mammalian nuclei Nucleic Acids Res 1983 11 1475 1489 10.1093/nar/11.5.1475 6828386 PMC325809

[B70] van der Deen M Akech J Lapointe D Gupta S Young DW Montecino MA Galindo M Lian JB Stein JL Stein GS van Wijnen AJ Genomic promoter occupancy of runt-related transcription factor RUNX2 in Osteosarcoma cells identifies genes involved in cell adhesion and motility J Biol Chem 2012 287 4503 4517 10.1074/jbc.M111.287771 22158627 PMC3281617

[B71] Langmead B Trapnell C Pop M Salzberg SL Ultrafast and memory-efficient alignment of short DNA sequences to the human genome Genome Biol 2009 10 R25 10.1186/gb-2009-10-3-r25 19261174 PMC2690996

[B72] Kent WJ Sugnet CW Furey TS Roskin KM Pringle TH Zahler AM Haussler D The human genome browser at UCSC Genome Res 2002 12 996 1006 10.1101/gr.229102 12045153 PMC186604

[B73] McLean CY Bristor D Hiller M Clarke SL Schaar BT Lowe CB Wenger AM Bejerano G GREAT improves functional interpretation of cis-regulatory regions Nat Biotechnol 2010 28 495 501 10.1038/nbt.1630 20436461 PMC4840234

