# Supplementary material for: Genomic occupancy of Runx2 with global expression profiling identifies a novel dimension to control of osteoblastogenesis
Source: Genome Biol. 2014 Mar 21;15(3):R52. doi: 10.1186/gb-2014-15-3-r52 (PMC4056528; doi:10.1186/gb-2014-15-3-r52)
Supplement: Additional file 1: Table S3 — Summary of MC3T3 Runx2 ChIP-Seq. This table lists the read numbers and genome coverage of Runx2 ChIP-Seq libraries. [file gb-2014-15-3-r52-S1.pdf]

Table S3 Summary of Runx2 ChIP-Seq libraries

Time point	Replicate	Raw reads	Peaks	Genome coverage
Day 0	2	77,753,556	25,457	6.22 x
Day 9	2	33,414,085	60,596	2.67 x
Day 28	2	28,418,309	40,330	2.27 x

Note: genome coverage = (number of raw reads number x average fragment size)/mouse genome size. Average fragment size = 200 bp, and mouse genome size = 2.5×10^9 bp.
