# Supplementary material for: Genomic occupancy of Runx2 with global expression profiling identifies a novel dimension to control of osteoblastogenesis
Source: Genome Biol. 2014 Mar 21;15(3):R52. doi: 10.1186/gb-2014-15-3-r52 (PMC4056528; doi:10.1186/gb-2014-15-3-r52)
Supplement: Additional file 2: Table S4 — Distribution patterns of Runx2 peaks across genomic locations. This table is related to Figure 2. [file gb-2014-15-3-r52-S2.pdf]

Table S4 Distribution patterns of Runx2 peaks across 6 categories of genomic locations

number of peaks at different stages of differentiation							
	**upstream**	**exon**	**intron**	**promoter**	**tts**	**intergenic**	total
proliferation (day 0)	2678	3470	8341	4490	229	6249	25457
matrix deposition (day9)	6665	7384	24646	5339	670	15892	60596
mineralization (day 28)	4345	4994	15072	5185	385	10349	40330

Fraction of Runx2 peaks at different stages of differentiation							
	**upstream**	**exon**	**intron**	**promoter**	**tts**	**intergenic**	total
proliferation (day 0)	10.5%	13.6%	32.8%	17.6%	0.9%	24.5%	100.0%
matrix deposition (day9)	11.0%	12.2%	40.7%	8.8%	1.1%	26.2%	100.0%
mineralization (day 28)	10.8%	12.4%	37.4%	12.9%	1.0%	25.7%	100.0%

number and fraction of all Runx2 peaks during differentiation							
	**upstream**	**exons**	**introns**	**promoters**	**tts**	**intergenic**	total
peak number	8661	9066	31598	7118	837	21461	78741
fraction	11.0%	11.5%	40.1%	9.0%	1.1%	27.3%	100.0%
random control	6.3% ± 0.06%	1.7% ± 0.02%	23.5% ± 0.08%	0.5% ± 0.04%	0.5% ± 0.03%	67.5% ± 0.07%	100.0%
