# Supplementary material for: Genomic occupancy of Runx2 with global expression profiling identifies a novel dimension to control of osteoblastogenesis
Source: Genome Biol. 2014 Mar 21;15(3):R52. doi: 10.1186/gb-2014-15-3-r52 (PMC4056528; doi:10.1186/gb-2014-15-3-r52)
Supplement: Additional file 3 — Detailed description of ChIP-PCR, ChIP-Seq with bioinformatics analysis, and supplemental figure legends. [file gb-2014-15-3-r52-S3.pdf]

Supplemental Materials and Methods

Chromatin immunoprecipitation and High-throughput sequencing

At day 0, 9, and 28 of differentiation, approximately $1x10^8$ MC3T3-E1 cells were washed with PBS and then fixed with 1% formaldehyde for 8 minutes to crosslink DNA-protein complexes. The fixed cells were washed with ice-cold PBS, harvested by scraping, pelleted, and resuspended in nuclei isolation buffer A (50 mM HEPES pH 7.5, 140 mM NaCl, 1 mM EDTA, 10% glycerol, 0.5% IGEPAL CA-630, 0.25% Triton-X100, Roche complete protease inhibitor) for 10 minutes at 4°C with constant rotation. Nuclei were then pelleted by centrifugation, washed in washing buffer B for 10 minutes at room temperature (10 mM Tris-HCl pH 8.0, 200 mM NaCl, 1 mM EDTA, 1 mM EGTA, Roche complete protease inhibitor), collected by centrifugation and resuspended in lysis buffer C (10 mM Tris-HCl pH 8.0, 100 mM NaCl, 1 mM EDTA, 1 mM EGTA, 0.1% sodium deoxycholate, 0.5% N-laurylsarcosine Roche complete protease inhibitor). Isolated chromatin was sheared using a Misonix S-4000 ultrasonic sonicator for 12 minutes (1 second on, 2 seconds off) at 30% power output to a range from 0.2 kb to 0.6 kb. Isolated chromatin was then used for immunoprecipitation with Runx2 antibody (M-70, Santa Cruz) or immunoglobulin G (IgG) (12-370, Millipore) as a control followed by Protein-G Dynabeads (Invitrogen). Immunoprecipitates were collected, washed three times with low salt buffer (20 mM Tris-HCl pH 8.0, 1 mM EDTA, 0.1% SDS, 1% Triton-X100, and 150 mM NaCl), washed one time with high salt buffer (20 mM Tris-HCl pH 8.0, 1 mM EDTA, 0.1% SDS, 1% Triton-X100, and 500 mM NaCl) and one time with TEN buffer (50 mM Tris-HCl pH 8.0, 1 mM EDTA, and 50 mM NaCl), and eluted into elution buffer (50 mM Tris-HCl pH 8.0, 10 mM EDTA, and 1% SDS) at 55°C for 30 minutes with shaking. Chromatin was then uncrosslinked at 65^oC overnight, and treated with RNase A for 2 hours followed by Proteinase K for 2 hours at 55°C. DNA was then recovered by phenol/chloroform/isoamyl alcohol (25:24:1) extraction followed by ethanol precipitation. Uncrosslinked ChIP DNA was then end-repaired using End-It DNA End-repair Kit (Epicentre Biotechnologies), A-tailed with Klenow DNA polymerase (Epicentre Biotechnologies)

and ligated to Illumina SR adapters (Illumina) using Fast-Link DNA ligase (Epicentre Biotechnologies). Adapter-ligated DNA libraries were then amplified by PCR and the resulting product resolved by agarose-gel electrophoresis. A fraction of DNA with inserted fragments of 200 ± 50 bp was then excised and gel purified. Purified DNA was sequenced for single-end 36 bases on an Illumina Genome Analyzer II following manufacturer's protocols. Base calls and sequence reads were generated by Illumina CASAVA software (version 1.6, Illumina). Two independent biological repeats of Runx2 ChIP-Seq libraries were prepared for each time point, and two input libraries were prepared with sonicated DNA from day 9 MC3T3-E1 cells. The detailed information on Runx2 ChIP-Seq libraries can be found in Additional file 2, Table S3.

ChIP-qPCR

For each ChIP-qPCR reaction, 0.2 ng DNA recovered from ChIP experiments were used as template. Fold enrichment of targeted regions was calculated by normalizing the enrichment of targeted regions in Runx2 ChIP to that in control IgG ChIP from three biological replicates using $2^{-\Delta\Delta C_T}$ method. Primers for ChIP-qPCR reactions were designed by FoxPrimer (www.foxprimer.org, Dobson, J.R., PhD thesis 2013 at UMASS Medical School), and are available in Additional file 15, Table S1.

Bioinformatics analysis of ChIP-Seq data

A. Analysis of ChIP-seq data

Short reads from two biological repeats of Runx2 ChIP-seq and input libraries were combined and mapped respectively to the mouse genome (assembly mm9) using Bowtie (version 0.12.8) [1]. Read densities and regions of statistically significant enrichment (“peaks”) were determined using MACS (Model-Based Analysis of ChIP-Seq, version 1.4.1) [2] using default settings. Peaks of ChIP-seq enrichment with $p < 10^{-10}$, relative to input, were considered significant and

included in subsequent analyses. During differentiation, 25,457 (day 0), 60,596 (day 9) and 40,330 (day 28) unique peaks were called. ChIP-seq read densities were normalized to a total of 10 million reads across each of the three time-points that were collected.

The RUNX2 motif (position weight matrix) was determined *de novo* using MEME-ChIP in MEME SUITE (version 4.7.0) [3] to analyze the 500 most significant peaks according to MACS in our ChIP-seq data-sets. The resulting motif was present in 459 out of 500 peaks with an E-value of 4.7×10^{-200}. The *de novo* motif was compared to a library of known motifs using TOMTOM [3] and was similar (E-value=2.6×10^{-4}, q-value=2.6×10^{-4}) to the JASPAR MA0002.2 RUNX motif [4]. To compute the distribution of RUNX2 motifs among categories of genomic locations, our *de novo* discovered RUNX2 motif was scanned across the mouse genome using FIMO [3] (version 4.7.0) with a significance threshold of $p < 10^{-4}$, resulting in 780,747 occurrences.

For the purposes of classifying Runx2 binding regions on the basis of genomic location categories, gene annotations were taken from RefSeq [5], and elements had the following definitions: promoters were 1000 bp upstream of TSSs to 150 bp downstream of TSSs, excluding any exons or introns; upstream elements 20 kb upstream of TSSs to 1000 upstream bp of TSSs, excluding any overlaps with promoters, exons or introns; TTS regions were 150 bp upstream of TTSs to 1000 bp downstream of TTSs, excluding any exons, introns or promoters and intergenic genomic regions were those that satisfy none of the above definitions. The distributions of ChIP-seq binding regions among categories of genomic locations were determined by locating each peak summit in one category.

A random background distribution among location categories, so defined, was computed by sampling genomic coordinates from a uniform distribution with size matched to the summit regions of Runx2 peaks (peak summit 50 ± bp). To estimate the variance in this distribution, the background was independently sampled five times, in each case for a number of “peaks” equal

to that in the Runx2 ChIP-seq data. Runx2 binding patterns at three differentiation stages were characterized to seven clusters based on the absence and presence of peaks.

B. GREAT analysis

GO term analyses of ChIP-Seq peaks in each cluster were done in GREAT (version 2.0.2, http://bejerano.stanford.edu/great/public/html) with its default association rule of peak to gene at 1 Mb [6]. All listed terms satisfy a binomial and hypergeometric FDR q-value ≤ 0.05 and binomial and hypergeometric fold enrichment ≥ 2. The blue boxes left to each term are indicative of scores of p value from binomial enrichment test (i.e., score = -log10(p value)). Term coverage is computed as: coverage = (number of gene associated with Runx2 peaks of a term)/(total number of genes in a term).

C. Runx2 binding profiles of Runx2 responsive genes

Runx2 binding profiles (average peak number, peak distribution across genomic location categories, and average fold change of peak mean signals) of shRunx2 responsive genes, upregulated or downregulated, were compared respectively to those aspects of control shRunx2 non-responsive genes characterized in Affymetrix microarray profiling at day 9. shRunx2 responsive genes are those mRNA levels significantly changed by shRunx2 treatment with a FDR threshold of 0.05 and a fold-change cutoff of 1.5 in comparison to that in control Scr treatment; those genes that did not meet these criteria, were defined as non-responsive genes to shRunx2. Average peak number was defined as the mean number of peaks per gene in a given group of genes. Peak numbers at day 0, day 9, and combined days 0 and 9, were compared between down- or up-regulated genes versus control. Statistical significance of the differences in peak numbers was determined by non-parametric Mann-Whitney test. Peak distribution patterns were obtained by partitioning all peaks associated with shRunx2 responsive and non-responsive genes at day 9 into genomic location categories. The statistical significance

of differences in distribution patterns between down- or up-regulated genes versus control was determined by Fisher's exact test. Average fold change of peak mean signals for any Runx2 peak at day 9 was defined as: average fold change = (mean signals of Runx2 peak covering a given genomic region at day 9/mean signal of the corresponding genomic region at day 0). Average fold change of peak mean signals were compared pairwise between down- or up-regulated genes versus control in each genomic location category. Non-parametric Mann-Whitney test was used to calculate the statistical significance of these comparisons.

D. PeaksToGenes

Another independent method based PeaksToGenes (https://github.com/peakstogenes/PeaksToGenes, Dobson J.R., PhD thesis 2013 at UMASS Medical School) was used for unbiased identification of genome-wide patterns between the positions of Runx2 binding events and genes responsive to shRunx2. For each gene in RefSeq mm9 [5], PeaksToGenes defined relative genomic coordinates. Regions flanking genes were defined as ten 1 kb non-overlapping intervals 5' and 3' of the transcription start site and transcription termination site, respectively. Within the gene body of each gene, two types of relative genomic coordinates were defined: 1) ten approximately equal non-overlapping intervals from TSS to TTS, and 2) non-overlapping coordinates derived from the genomic positions of 5'-UTRs, introns, exons, and 3'-UTRs for the primary gene transcript. Therefore, for each gene in RefSeq mm9, 34 relative genomic coordinates were defined. Within each relative genomic coordinate for each gene, PeaksToGenes calculated the read-normalized ratio [7] of Runx2 ChIP-seq reads to Input reads for each day of MC3T3 differentiation. Using the limma TopTable results for the comparison between shRunx2 and Scramble shRNA on day 9, genes significantly responsive to shRunx2 were defined as having an adjusted p-value less than 0.05 and a fold-change greater than or less than 1.5 for upregulated and downregulated genes, respectively. Transcripts in the Mouse Gene 1.0ST Array Rev 4 (Affymetrix), which did not meet these

criteria, were defined as non-responsive to shRunx2. To calculate the probability that the distribution of Runx2 binding is the same in regions near responsive and non-responsive genes, in each relative genomic region defined above a comparison between the distribution of Runx2 binding events for responsive and non-responsive genes was made. Using a non-parametric Wilcoxon rank sum test [8], PeaksToGenes compared the distributions of Runx2 enrichment ratios (IP/input) between upregulated genes and non-responsive as well as between downregulated and non-responsive genes.

E. EMBER

Correlation analysis of Runx2 binding and Runx2 responsive genes were done by EMBER [9]. Given a set of TF binding regions and relevant expression data collected under a series of conditions, EMBER uses expectation maximization to deduce an expression matrix, $S_{l,m}$, and use it to assign regulatory target genes. Each gene x_i is given a score,

$$S(x_i) = \sum_{l=1}^{L} \sum_{m=--}^{++} S_{l,m} \delta_{m, x_i, l}, \quad (1)$$

where $S_{l,m} = \log(f_{l,m}^{\text{model}} / f_{l,m}^{\text{background}})$ defines the score matrix. The behavior dimensions, l, are for each comparison that is made using expression data. In our case, L = 2. The number possible classifications, m, allowed for each behavior dimension is M. Following Maienschein-Cline et al. [9], we use M = 5 with m = (−−, −, 0, +, ++), where −− means that a large down-regulation was observed: 3s < μ1 − μ2, where s = σ2 + σ2 is the sum of standard deviations between normalized expression levels of gene xi. A small (−) downregulation is s < μ1 − μ2 ≤ 3s and so on. Genes are taken as potential regulatory targets of a binding locus if they lie within 100 kb of it, implying many possible regulatory targets for each binding locus.

References

1. Langmead B, Trapnell C, Pop M, Salzberg SL: **Ultrafast and memory-efficient alignment of short DNA sequences to the human genome.** *Genome Biol* 2009, **10**:R25.
2. Zhang Y, Liu T, Meyer CA, Eeckhoute J, Johnson DS, Bernstein BE, Nusbaum C, Myers RM, Brown M, Li W, Liu XS: **Model-based analysis of ChIP-Seq (MACS).** *Genome Biol* 2008, **9**:R137.
3. Bailey TL, Boden M, Buske FA, Frith M, Grant CE, Clementi L, Ren J, Li WW, Noble WS: **MEME SUITE: tools for motif discovery and searching.** *Nucleic Acids Res* 2009, **37**:W202-208.
4. Portales-Casamar E, Thongjuea S, Kwon AT, Arenillas D, Zhao X, Valen E, Yusuf D, Lenhard B, Wasserman WW, Sandelin A: **JASPAR 2010: the greatly expanded open-access database of transcription factor binding profiles.** *Nucleic Acids Res* 2010, **38**:D105-110.
5. Pruitt KD, Tatusova T, Maglott DR: **NCBI reference sequences (RefSeq): a curated non-redundant sequence database of genomes, transcripts and proteins.** *Nucleic Acids Res* 2007, **35**:D61-65.
6. McLean CY, Bristor D, Hiller M, Clarke SL, Schaar BT, Lowe CB, Wenger AM, Bejerano G: **GREAT improves functional interpretation of cis-regulatory regions.** *Nat Biotechnol* 2010, **28**:495-501.
7. Landt SG, Marinov GK, Kundaje A, Kheradpour P, Pauli F, Batzoglou S, Bernstein BE, Bickel P, Brown JB, Cayting P, Chen Y, DeSalvo G, Epstein C, Fisher-Aylor KI, Euskirchen G, Gerstein M, Gertz J, Hartemink AJ, Hoffman MM, Iyer VR, Jung YL, Karmakar S, Kellis M, Kharchenko PV, Li Q, Liu T, Liu XS, Ma L, Milosavljevic A, Myers RM*, et al.*: **ChIP-seq guidelines and practices of the ENCODE and modENCODE consortia.** *Genome Res* 2012, **22**:1813-1831.
8. Wilcoxon F: **Individual comparisons by ranking methods.** *Biometrics Bull* 1945, **1**:80-83.

9. Maienschein-Cline M, Zhou J, White KP, Sciammas R, Dinner AR: **Discovering transcription factor regulatory targets using gene expression and binding data.** *Bioinformatics* 2012, **28**:206-213.

Supplemental Figure Legends

Figure S1 Top GO terms assigned by GREAT analysis for clusters 2, 3, 5, and 7 in Figure 3A. Assigned terms from each cluster were ranked by the enrichment score (-$\log_{10}$(p value from binomial enrichment test)) and satisfy binomial and hypergeometric FDR q-value ≤ 0.05 and binomial and hypergeometric fold enrichment ≥ 2. Values to the right side of each term are the coverage (number of genes associated with Runx2 peaks / total number of genes) of the corresponding term. Abbreviations: neg., negative; pos., positive; reg., regulation of; morpho., morphogenesis; dev., development; RSKs, Threonine/serine kinases. Detailed information of the genes in each term is available in Additional file 1, Table S5.

Figure S2 Runx2 binding enriches at *Bsp* locus during the progression of osteogenic differentiation. Gene annotation follows standard gene prediction display conventions used by UCSC genome browser (exons: solid boxes; introns: solid lines; direction of gene transcription: arrows). Positions of Runx2 peaks called by MACS (green bars) and Runx2 consensus motif (TGTGGT) (solid black bar) are also depicted. Input track (light blue) was included for visualizing background noise during peak calling.

Figure S3 Verification of Runx2 knockdown by shRunx2 in MC3T3 cells. (A) Alkaline phosphatase staining of cells from Runx2 shRNA (shRunx2) knockdown and scramble shRNA control (Scr). Runx2 shRNA clearly decreased alkaline phosphatase staining. **(B)** Expression of osteogenic marker *Ocn*, *Bsp*, *Akp2*, *Osx*, *Col1a1*, *Runx2* upon Runx2 knockdown (shRunx2), when compared with scramble control (Scr). 5 out of 6 markers showed significant decrease in mRNA level upon Runx2 knockdown (*: $p< 0.01$, **: $p < 0.05$, *t*-test). **(C)** Runx2 protein level decreased by 80% upon Runx2 shRNA treatment (shRunx2), in contrast to scramble shRNA control (Scr).

Figure S4 Additional characteristics of Runx2 binding in shRunx2 responsive genes. (A) Average gene length of shRunx2 responsive genes (downregulated (Down) and upregulated (Up)), in contrast non-responsive genes. Downregulated genes are significantly longer ($p < 0.05$) than control and upregulated genes. **(B)** shRunx2 downregulated genes (Down) associate with more Runx2 peaks than upregulated (Up), Non-responsive, and length-matched control genes (Non-responsive Down); whereas shRunx2 upregulated genes associate with less Runx2 peaks than control. **(C)** shRunx2 upregulated genes have significantly less peaks than Non-responsive and length-matched (Non-responsive Up) controls. **(D)** Downregulated genes have more intronic peaks than shRunx2 upregulated genes and Non-responsive control, whereas upregulated genes have less intronic peaks. Responsive genes were differentially expressed at FDR threshold of 0.05 and fold change cutoff of 1.5. Comparisons were performed between shRunx2 responsive genes and gene length-matched sets of randomly selected non-responsive genes: Non-responsive Down genes for downregulated genes, and Non-responsive Up genes for upregulated genes. In (B-D), Runx2 peaks used in the analysis were combined from days 0, 9, and 28 datasets. Asterisks (*) indicate statistical significance ($p < 0.05$) calculated using Kolmogorov-Smirnov test.

Figure S5 PeaksToGenes analysis of Runx2 occupancy in Runx2 shRNA responsive genes. (A) Profile of Runx2 binding at gene bodies and flanking 10 kb regions in proliferating (day 0) and matrix depositing (day 9) MC3T3-E1 cells. K-means clustering was used to generate five distinct clusters from Runx2 peaks: I: moderate promoter and strong gene body binding; II: strong promoter, moderate gene body and flanking 10 kb binding; III: strong promoter and moderate flanking 10 kb binding; IV: moderate binding over all regions; V: weak binding across all regions. **(B)** Table of distribution of upregulated and downregulated genes ($\geq$ 1.5 fold change, FDR $\leq$ 0.05) by shRunx2 in the five clusters (determined in A). Numbers and fraction of genes (in parentheses) in each cluster are shown. Statistical significance was

determined by Fisher’s exact test: upregulated versus non-responsive ($p = 0.2397$); downregulated versus non-responsive ($p < 0.0001$); upregulated versus downregulated ($p = 0.0001$).

Figure S6 EMBER analyses of Runx2 binding in the genes differentially regulated by Runx2 knockdown. Expression levels of genes differentially regulated by shRunx2 were grouped into five categories: strongly upregulated (++), upregulated (+), not changed (0), downregulated (-), and strongly downregulated (--). The expression patterns were defined by EMBER based on both gene expression levels and Runx2 binding at gene body ± 100kb. Expression patterns from 42 groups of Runx2 binding regions associated with differentially expressed genes (7 clusters x 6 genomic location categories) were analyzed, resulting in 42 score matrices. The EMBER score matrices of each expression pattern were depicted as bars of length 3, analogous to sequence motif logos. The heights of bars (Bits) indicate the score determined by EMBER matrices, measuring the amount of gene expression that can be explained by Runx2 binding compared to control (background). Results from EMBER suggested that peaks in cluster 4 (days 9 & 28) and cluster 6 (day 9) located in specific genomic regions (upstream, promoter, exons, and intergenic) associate with gene expression more than peaks located in intronic and TTS regions. Cluster 1: ubiquitous; cluster 2: days 0 & 28; cluster 3: days 0 & 9; cluster 4: days 9 & 28; cluster 5: day 28; cluster 6: day 9; cluster 7: day 0.

Figure S7 Validation of novel Runx2 target *Tnfrsf19*. (A) *Tnfrsf19* mRNA expression increases upon differentiation. **(B)** Runx2 knockdown (shRunx2) significantly decreases (*: $p < 0.001$) the expression of *Tnfrsf19* in MC3T3-E1 cells. **(C)** Runx2 binding at *Tnfrsf19* during osteoblastogenesis. Gene annotation follows standard gene prediction display conventions used by UCSC genome browser (exons: solid boxes; introns: solid lines; direction of gene transcription: arrows). Positions of Runx2 peaks called by MACS (green bars) and Runx2

consensus motif (TGTGGT) (solid black bar) are also depicted. Input track (light blue) was included for visualizing background noise during peak calling. All values in the panel (A) and (B) are mean ± SEM from three biological replicates of experiments.

Figure S8 ChIP-qPCR validation of Runx2 peaks identified by MACS. (A) Runx2 peaks from nine genes (*Runx2*, *Bglap2/Ocn*, *Ibsp/Bsp*, *Ezh2*, *Adamts4*, *Crabp2*, *Stat1*, *Tnfrsf19*, *Smad4*), together with two negative controls (Negative control 1 and *Twist2* promoter) were amplified by ChIP-qPCR with Runx2 or control IgG antibody from day 9 differentiated MC3T3-E1 cells. For each ChIP-qPCR reaction, 0.2 ng DNA from Runx2 or IgG ChIP were used. The values represented in the histograms are mean fold enrichment in Runx2 ChIP samples over IgG ChIP samples from 3 biological replicates, and error bars are S.E.M. Statistical significance of the differences in fold enrichment was determined by *t*-test. *: $p < 0.01$; **: $p < 0.05$. The PCR-amplified genomic locations reside in the blue boxes of Runx2 occupancy tracks in each panel, and the green bars demarcate the peaks called by MACS. The detailed information of the primers is included in Additional File 15 Table S1.
