# Supplementary material for: Genomic occupancy of Runx2 with global expression profiling identifies a novel dimension to control of osteoblastogenesis
Source: Genome Biol. 2014 Mar 21;15(3):R52. doi: 10.1186/gb-2014-15-3-r52 (PMC4056528; doi:10.1186/gb-2014-15-3-r52)
Supplement: Additional file 5: Figure S1 — GO term analysis from GREAT for clusters 1 to 3 and 5 to 7 in Figure 3A. This is related to Figure 3. [file gb-2014-15-3-r52-S5.pdf]

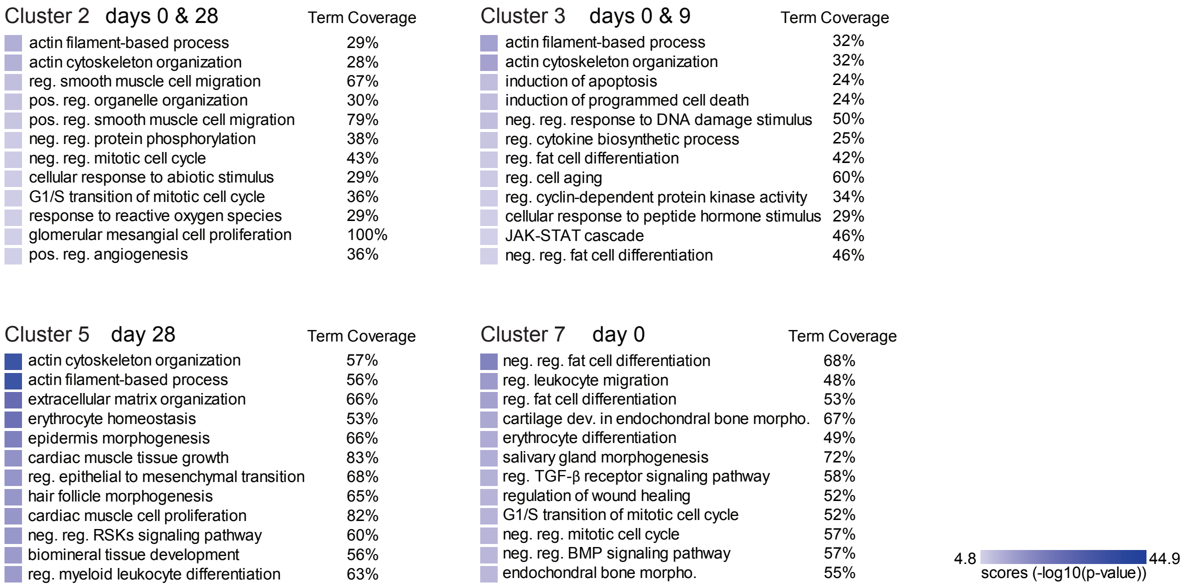

Figure S1. Top GO terms assigned by GREAT analysis for clusters 2, 3, 5, and 7 in Figure 3A. Assigned terms from each cluster were ranked by the enrichment score ($-\log_{10}$(p value from binomial enrichment test)) and satisfy binomial and hypergeometric FDR q-value ≤ 0.05 and binomial and hypergeometric fold enrichment ≥ 2. Values to the right side of each term are the coverage (number of genes associated with Runx2 peaks / total number of genes) of the corresponding term. Abbreviations: neg., negative; pos., positive; reg., regulation of; morpho., morphogenesis; dev., development; RSKs, Threonine/serine kinases. Detailed information of the genes in each term is available in Additional file 4, Table S5.
