# Supplementary material for: Genomic occupancy of Runx2 with global expression profiling identifies a novel dimension to control of osteoblastogenesis
Source: Genome Biol. 2014 Mar 21;15(3):R52. doi: 10.1186/gb-2014-15-3-r52 (PMC4056528; doi:10.1186/gb-2014-15-3-r52)
Supplement: Additional file 6: Figure S8 — Validation of Runx2 peaks by ChIP-PCR. This figure is related to Figures 4, 5 and 6. [file gb-2014-15-3-r52-S6.pdf]

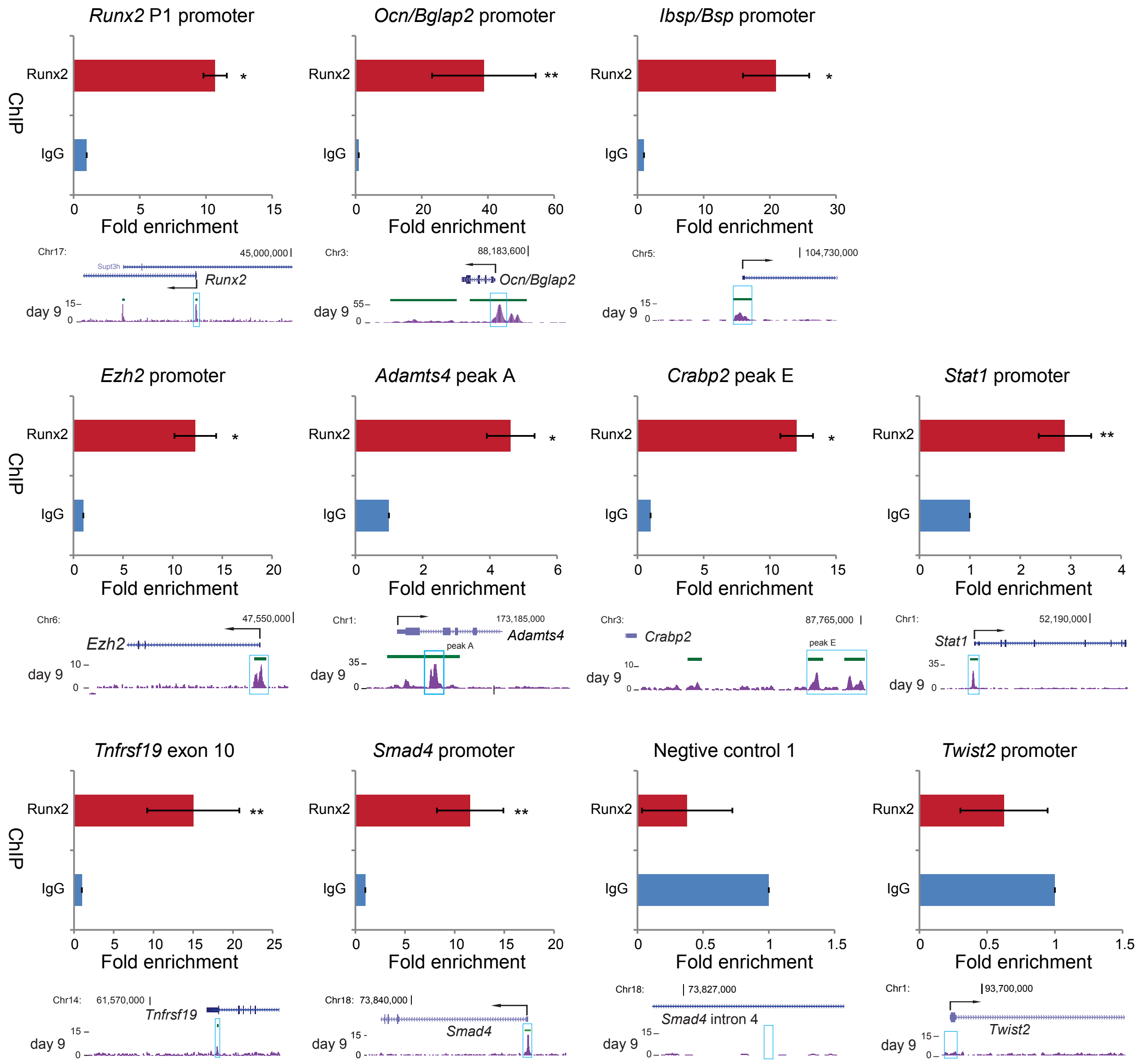

Figure S8 ChIP-qPCR validation of Runx2 peaks identified by MACS. Runx2 peaks from nine genes (*Runx2*, *Bglap2/Ocn*, *Ibsp/Bsp*, *Ezh2*, *Adamts4*, *Crabp2*, *Stat1*, *Tnfrsf19*, *Smad4*), together with two negative controls (Negative control 1 and *Twist2* promoter) were amplified by ChIP-PCR with Runx2 or control IgG antibody from day 9 differentiated MC3T3-E1 cells. For each ChIP-qPCR reaction, 0.2 ng DNA from Runx2 or IgG ChIP were used. The values represented in the histograms are mean fold enrichment in Runx2 ChIP samples over IgG ChIP samples from 3 biological replicates, and error bars are S.E.M. Statistical significance of the differences in fold enrichment was determined by t-test. *: $p < 0.01$; **: $p < 0.05$. The PCR-amplified genomic locations reside in the blue boxes of Runx2 occupancy tracks in each paneland the green bars demarcate the peaks called by MACS. The detailed information of the primers is included in Additional File 15 Table S1.
