# Supplementary material for: Genomic occupancy of Runx2 with global expression profiling identifies a novel dimension to control of osteoblastogenesis
Source: Genome Biol. 2014 Mar 21;15(3):R52. doi: 10.1186/gb-2014-15-3-r52 (PMC4056528; doi:10.1186/gb-2014-15-3-r52)
Supplement: Additional file 7: Figure S2 — Runx2 peaks associated with Bsp gene during differentiation. This figure is related to Figure 4. [file gb-2014-15-3-r52-S7.pdf]

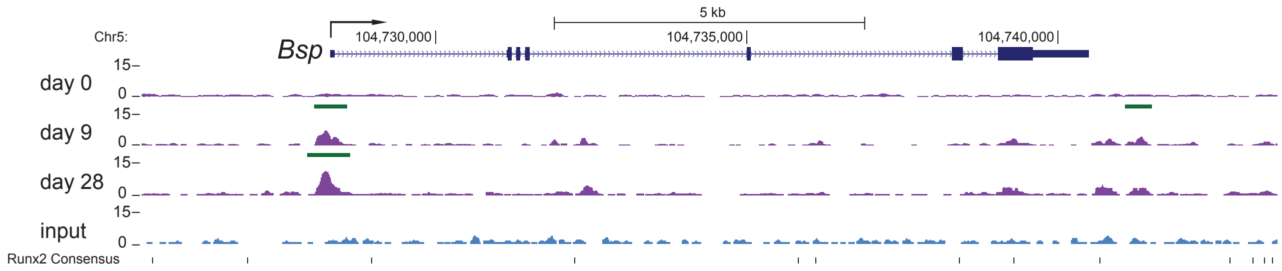

Figure S2. Runx2 binding enriches at Bsp locus during the progression of osteogenic differentiation. Gene annotation follows standard gene prediction display conventions used by UCSC genome browser (exons: solid boxes; introns: solid lines; direction of gene transcription: arrows). Positions of Runx2 peaks called by MACS (green bars) and Runx2 consensus motif (TGTGGT) (vertical black lines) are also depicted. Input track (light blue) was included for visualizing background noise during peak calling.
