# Supplementary material for: Genomic occupancy of Runx2 with global expression profiling identifies a novel dimension to control of osteoblastogenesis
Source: Genome Biol. 2014 Mar 21;15(3):R52. doi: 10.1186/gb-2014-15-3-r52 (PMC4056528; doi:10.1186/gb-2014-15-3-r52)
Supplement: Additional file 9: Figure S3 — Validation of Runx2 knockdown in MC3T3 cells. This figure is related to Figure 5. [file gb-2014-15-3-r52-S9.pdf]

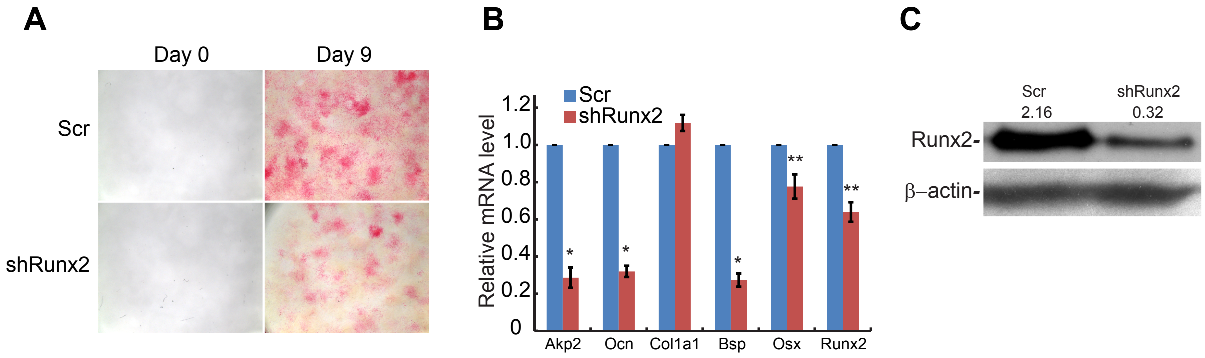

Figure S3. Verification of Runx2 knockdown by shRunx2 in MC3T3 cells. (A) Alkaline phosphatase staining of cells from Runx2 shRNA (shRunx2) knockdown and scramble shRNA control (Scr). Runx2 shRNA clearly decreased alkaline phosphatase staining. **(B)** Expression of osteogenic marker *Ocn*, *Bsp*, *Akp2*, *Osx*, *Col1a1*, *Runx2* upon Runx2 knockdown (shRunx2), when compared with scramble control (Scr). 5 out of 6 markers showed significant decrease in mRNA level upon Runx2 knockdown (*: $p < 0.01$, **: $p < 0.05$, t-test). **(C)** Runx2 protein level decreased by 80% upon Runx2 shRNA treatment (shRunx2), in contrast to scramble shRNA control (Scr).
