# Supplementary material for: Genomic occupancy of Runx2 with global expression profiling identifies a novel dimension to control of osteoblastogenesis
Source: Genome Biol. 2014 Mar 21;15(3):R52. doi: 10.1186/gb-2014-15-3-r52 (PMC4056528; doi:10.1186/gb-2014-15-3-r52)
Supplement: Additional file 10: Figure S4 — Additional characteristics of Runx2 binding in shRunx2 responsive genes. This figure is related to Figure 5. [file gb-2014-15-3-r52-S10.pdf]

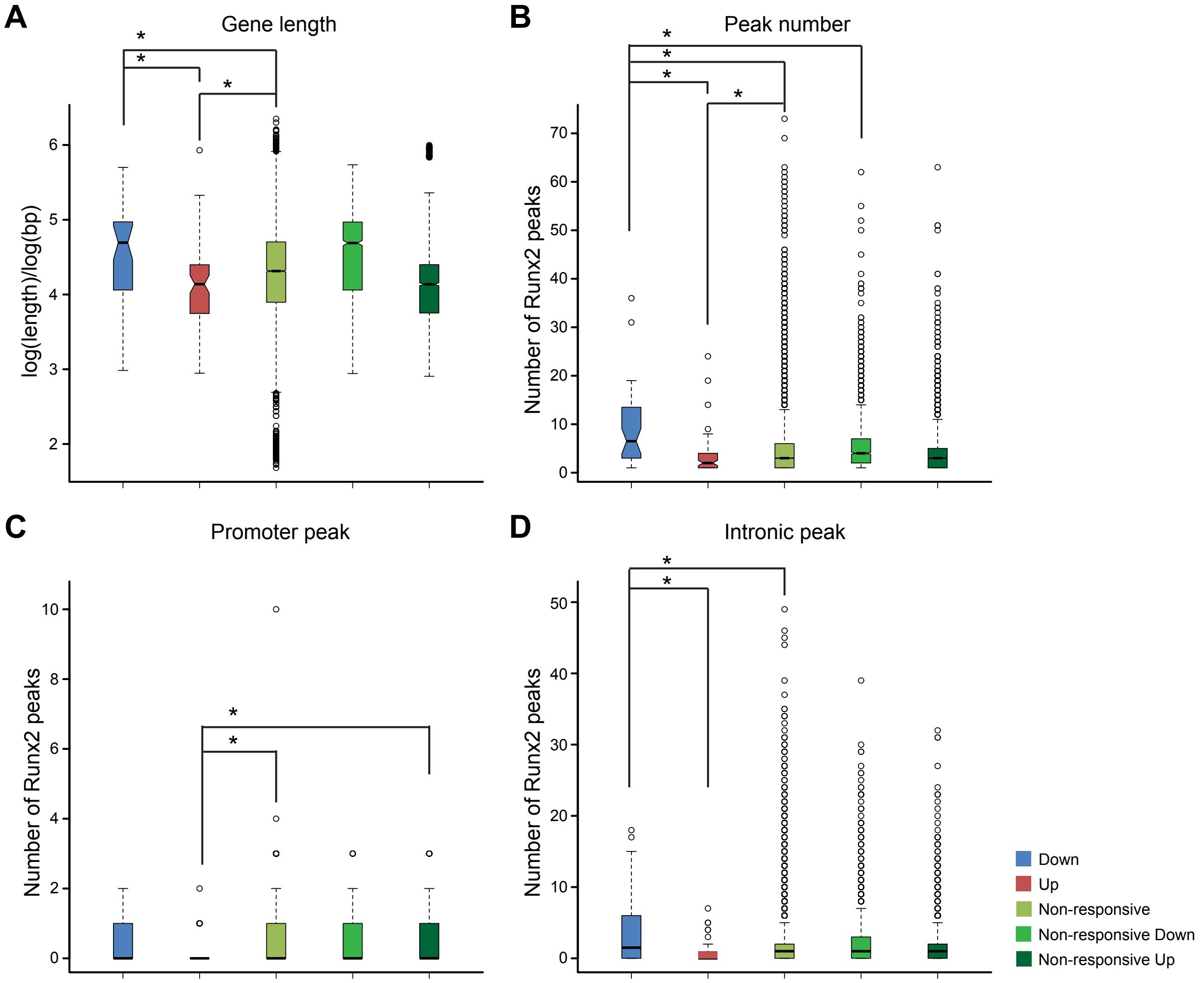

Figure S4. Additional characteristics of Runx2 binding in shRunx2 responsive genes. (A) Average gene length of shRunx2 responsive genes (downregulated (Down) and upregulated (Up)), in contrast to non-responsive genes. Downregulated genes are significantly longer ($p < 0.05$) than control and upregulated genes. **(B)** shRunx2 downregulated genes (Down) associate with more Runx2 peaks than upregulated (Up), Non-responsive, and length-matched control genes (Non-responsive Down); whereas shRunx2 upregulated genes associate with less Runx2 peaks than control. **(C)** shRunx2 upregulated genes have significantly less peaks than Non-responsive and length-matched (Non-responsive Up) controls. **(D)** Downregulated genes have more intronic peaks than shRunx2 upregulated genes and Non-responsive control, whereas upregulated genes have less intronic peaks. Responsive genes were differentially expressed at FDR threshold of 0.05 and fold change cutoff of 1.5. Comparisons were performed between shRunx2 responsive genes and gene length-matched sets of randomly selected non-responsive genes: Non-responsive Down genes for downregulated genes, and Non-responsive Up genes for upregulated genes. In (B-D), Runx2 peaks used in the analysis were combined from days 0, 9, and 28 datasets. Asterisks (*) indicate statistical significance ($p < 0.05$) calculated using Kolmogorov-Smirnov test.
