# Supplementary material for: Genomic occupancy of Runx2 with global expression profiling identifies a novel dimension to control of osteoblastogenesis
Source: Genome Biol. 2014 Mar 21;15(3):R52. doi: 10.1186/gb-2014-15-3-r52 (PMC4056528; doi:10.1186/gb-2014-15-3-r52)
Supplement: Additional file 11: Figure S5 — PeaksToGenes analysis of Runx2 occupancy in Runx2 shRNA-responsive genes. This figure is related to Figure 5E. [file gb-2014-15-3-r52-S11.pdf]

A

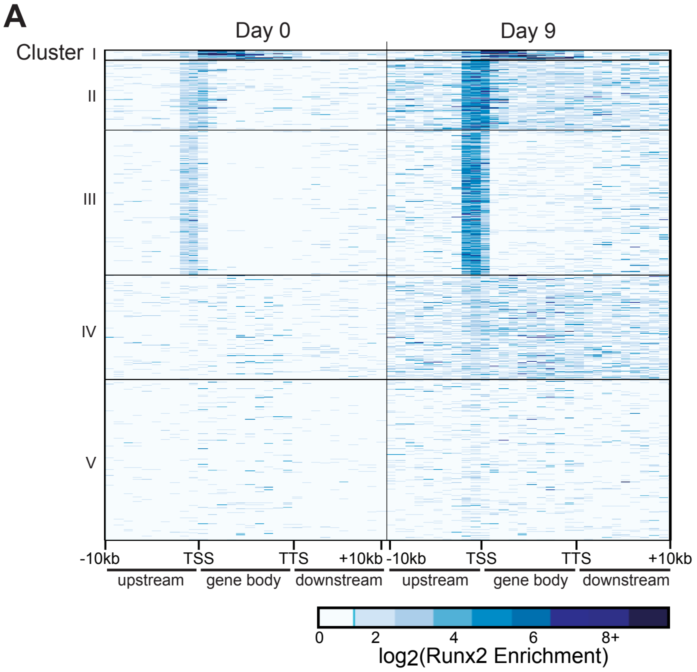

B

Number of peaks in different clusters				
Runx2 binding intensities	Cluster	non-responsive Genes	Upregulated Genes	Downregulated Genes
moderate promoter, strong genebody	I	350 (1.8%)	2 (1.7%)	3 (6.8%)
strong promoter, and moderate genebody and flanking10 kb	II	3214 (16.1%)	5 (4.3%)	6 (13.6%)
strong promoter and moderate flanking 10 kb binding	III	6195 (31.0%)	9 (7.8%)	13 (29.5%)
moderate Runx2 binding over all regions	IV	4038 (20.2%)	23 (20.0%)	9 (20.5%)
weak Runx2 binding across all regions	V	6168 (30.9%)	76 (66.1%)	13 (29.5%)
	Tota	19965	115	44

Figure S5. PeaksToGenes analysis of Runx2 occupancy in Runx2 shRNA responsive genes. (A) Profile of Runx2 binding at gene bodies and flanking 10 kb regions in proliferating (day 0) and matrix depositing (day 9) MC3T3-E1 cells. K-means clustering was used to generate five distinct clusters from Runx2 peaks: **I**: moderate promoter and strong gene body binding; **II**: strong promoter, moderate gene body and flanking 10 kb binding; **III**: strong promoter and moderate flanking 10 kb binding; IV: moderate binding over all regions; V: weak binding across all regions. **(B)** Table of distribution of upregulated and downregulated genes (≥ 1.5 fold change, FDR ≤ 0.05) by shRunx2 in the five clusters (determined in A). Numbers and fraction of genes (in parentheses) in each cluster are shown. Statistical significance was determined by Fisher's exact test: upregulated versus non-responsive ($p = 0.2397$); downregulated versus non-responsive ($p < 0.0001$); upregulated versus downregulated ($p = 0.0001$).
