# Supplementary material for: Genomic occupancy of Runx2 with global expression profiling identifies a novel dimension to control of osteoblastogenesis
Source: Genome Biol. 2014 Mar 21;15(3):R52. doi: 10.1186/gb-2014-15-3-r52 (PMC4056528; doi:10.1186/gb-2014-15-3-r52)
Supplement: Additional file 12: Figure S6 — EMBER analyses of Runx2 binding in the genes differentially regulated by Runx2 knockdown. This figure is related to Figure 5. [file gb-2014-15-3-r52-S12.pdf]

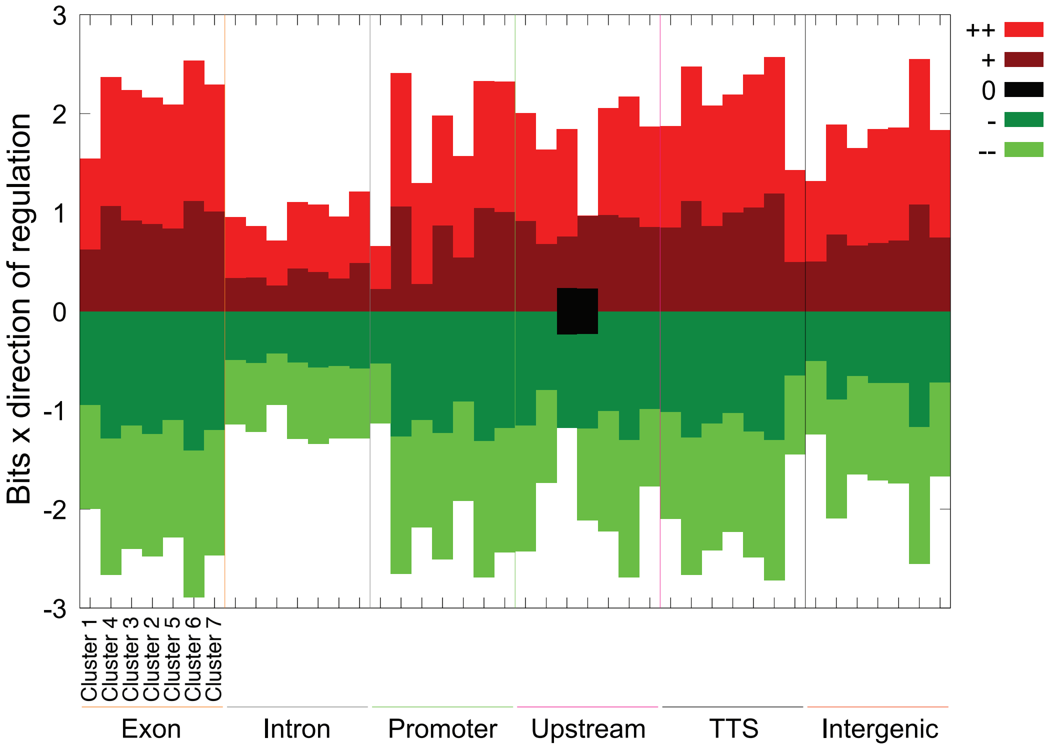

Figure S6. EMBER analyses of Runx2 binding in the genes differentially regulated by Runx2 knockdown. Expression levels of genes differentially regulated by shRunx2 were grouped into five categories: strongly upregulated (++), upregulated (+), not changed (0), downregulated (-), and strongly downregulated (--). The expression patterns were defined by EMBER based on both gene expression levels and Runx2 binding in the gene body ± 100kb. Expression patterns from 42 groups of Runx2 binding regions associated with differentially expressed genes (7 clusters x 6 genomic location categories) were analyzed, resulting in 42 score matrices. The EMBER score matrices of each expression pattern were depicted as bars of length 3, analogous to sequence motif logos. The heights of bars (Bits) indicate the score determined by EMBER matrices, measuring the amount of gene expression that can be explained by Runx2 binding compared to control (background). Results from EMBER suggested that peaks in cluster 4 (days 9 & 28) and cluster 6 (day 9) located in specific genomic regions (exons, promoter, upstream, TTS regions, and intergenic regions) associate with gene expression more than the peaks located in intronic regions. Cluster 1: ubiquitous; cluster 2: days 0 & 28; cluster 3: days 0 & 9; cluster 4: days 9 & 28; cluster 5: day 28; cluster 6: day 9; cluster 7: day 0.
