# Supplementary material for: Genomic occupancy of Runx2 with global expression profiling identifies a novel dimension to control of osteoblastogenesis
Source: Genome Biol. 2014 Mar 21;15(3):R52. doi: 10.1186/gb-2014-15-3-r52 (PMC4056528; doi:10.1186/gb-2014-15-3-r52)
Supplement: Additional file 13: Figure S7 — Validation of novel Runx2 target Tnfrsf19. This figure is related to Figure 6. [file gb-2014-15-3-r52-S13.pdf]

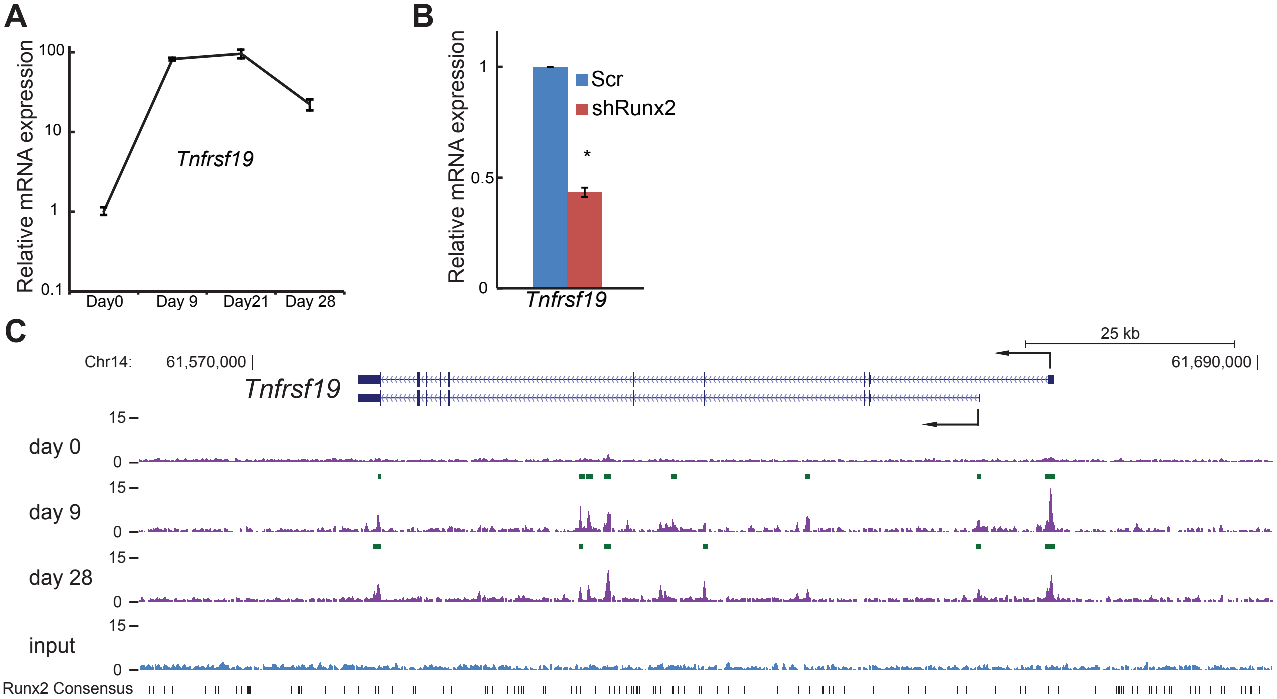

Figure S7. Validation of novel Runx2 target *Tnfrsf19*. (A) *Tnfrsf19* mRNA expression increases upon differentiation. **(B)** Runx2 knockdown (shRunx2) significantly decreases (*: $p < 0.001$) the expression of *Tnfrsf19* in MC3T3-E1 cells. **(C)** Runx2 binding at *Tnfrsf19* during osteoblastogenesis. Gene annotation follows standard gene prediction display conventions used by UCSC genome browser (exons: solid boxes; introns: solid lines; direction of gene transcription: arrows). Positions of Runx2 peaks called by MACS (green bars) and Runx2 consensus motif (TGTGGT) (solid black bar) are also depicted. Input track (light blue) was included for visualizing background noise during peak calling. All values in the panel (A) and (B) are mean ± SEM from three biological replicates of experiments.
