# Supplementary material for: Genomic occupancy of Runx2 with global expression profiling identifies a novel dimension to control of osteoblastogenesis
Source: Genome Biol. 2014 Mar 21;15(3):R52. doi: 10.1186/gb-2014-15-3-r52 (PMC4056528; doi:10.1186/gb-2014-15-3-r52)
Supplement: Additional file 16: Table S2 — Cloning primers. This table contains the primers used for plasmid construction. [file gb-2014-15-3-r52-S16.pdf]

Table S2 Cloning primers

Target gene / peak region	Forward primer	Reverse primer	Size (bp)
Adamts4 / Peak A	AACACGCGTCCCTACTGACCCCTATTCAC	GTCTCGAGTAGGGCTGGGAAGGAAGATA	1837
Adamts4 / Peak B	AACACGCGTGGGTTTATTCAGCTTGCACT	GTCTCGAGAACTAATGCTCAAAGATGCGGGGC	1540
Crabp2 / Peak C	AACACGCGTTGTATACTGAATGCATGCCT	GTAGATCTTCAGAGACCCAGCCTTTGTCATTGT	1114
Crabp2 / Peak D	AACACGCGTACCACCACCCAAAACAACTC	GTAGATCTGTAGACTGCTGGCCTTGACCTCAC	1103
Crabp2 / Peak E	AACACGCGTCTTTTTCTTATTCTTTGGAAC	GTAGATCTAACAAGAGGGAGAGGAAGAG	2192
